# Minimally Invasive, Bioadaptive Multimodal Sensor Probe with Safe Deployment for Real‐Time Acute Compartment Syndrome Diagnosis

**DOI:** 10.1002/advs.202506942

**Published:** 2025-07-17

**Authors:** Seung Gi Seo, Seungyeob Kim, Seonggwang Yoo, Seyong Oh, Haiwen Luan, Zengyao Lv, Bosung Kim, Shupeng Li, Di Lu, Jong Uk Kim, Yaeshin Park, Jae Hee Lee, Hyeon Bin Jo, Amanda M. Westman, William Moritz, Joseph Ribaudo, Yonggang Huang, Mitchell A. Pet, Sung Hun Jin, John A. Rogers

**Affiliations:** ^1^ Center for Bio‐Integrated Electronics Northwestern University Evanston IL 60208 USA; ^2^ Querrey Simpson Institute for Bioelectronics Northwestern University Evanston IL 60208 USA; ^3^ School of Electrical Engineering Korea Advanced Institute of Science & Technology Daejeon 34141 Republic of Korea; ^4^ College of Biomedical Science & Health Inje University Gimhae 50834 Republic of Korea; ^5^ Department of Digital Anti‐aging Healthcare Graduate School of Inje University Gimhae 50834 Republic of Korea; ^6^ Division of Electrical Engineering Hanyang University ERICA Ansan 15588 Republic of Korea; ^7^ Department of Mechanical and Aerospace Engineering University of California San Diego La Jolla CA 92093 USA; ^8^ Department of Civil and Environmental Engineering Northwestern University Evanston IL 60208 USA; ^9^ School of Microelectronics University of Science and Technology of China Hefei Anhui 230026 China; ^10^ Department of Information Display Kyung Hee University Seoul 02447 Republic of Korea; ^11^ Division of Plastic and Reconstructive Surgery Department of Surgery Washington University School of Medicine St. Louis MO 63110 USA; ^12^ Department of Mechanical Engineering Northwestern University Evanston IL 60208 USA; ^13^ Department of Materials Science and Engineering Northwestern University Evanston IL 60208 USA; ^14^ Department of Biomedical Engineering Northwestern University Evanston IL 60208 USA

**Keywords:** acute compartment syndrome diagnosis, multimodal sensor, minimally invasive, wireless operation

## Abstract

Acute Compartment Syndrome (ACS) is a serious medical condition that arises from increased pressure within osteofascial compartments, leading to impaired blood flow and potential tissue damage. Early and accurate diagnosis is critical for preventing permanent damage. Current methods rely largely on qualitative assessments with limited accuracy, and those that exploit invasive pressure measurements often prove inadequate. Herein, a soft materials‐based multimodal sensor probe is introduced, as well as the mechanical and thermal influences to monitor intra‐compartmental pressure, tissue oxygen saturation (StO_2_), and blood flow simultaneously at a common location within an affected compartment. The system integrates three sensors into a thin, flexible probe capable of real‐time, wireless data transmission. The device allows for continuous monitoring with high reproducibility and sensitivity, to enhance diagnostic accuracy relative to current clinical practice, with the potential to early diagnosis of an acute compartment syndrome that requires fasciotomies. Large animal model studies, including short‐ and intermediate‐term reliability assessments, highlight the key engineering features. The results reveal expected inverse relationships between pressure, StO_2_, and flow rate under simulated compartment syndrome conditions. This multimodal approach enhances diagnostic precision, offers real‐time insights, and promises to yield improved outcomes through a comprehensive, quantitative diagnosis of compartment syndrome.

## Graphical abstract

A soft, minimally invasive multimodal sensor probe integrates intracompartmental pressure, tissue oxygen saturation, and blood flow sensors into a single platform. By enabling precise measurements and capturing correlations among pressure, oxygenation, and vascular dynamics, the system provides a comprehensive physiological assessment, thereby enhancing diagnostic accuracy and facilitating the early detection of compartment syndrome.

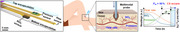

## 1 Introduction

Acute Compartment Syndrome (ACS) is a severe and potentially limb‐threatening condition that arises from increased pressure within a closed osteofascial compartment, leading to compromised blood flow and tissue perfusion.^[^ ^1^, ^2^ ^]^ Without prompt diagnosis and treatment, ACS can result in irreversible muscle and nerve damage. Conventionally, physicians diagnose ACS based only on qualitative clinical assessments and invasive intra‐compartmental pressure measurements, both of which can yield inconclusive results, even when taken together.^[^ ^3^, ^4^ ^]^ Symptoms such as pain, paraesthesia, and paralysis often present too late in the disease progression, resulting in high rates of missed diagnoses. Additionally, vascular signs, such as absent pulses and pallor, are often present only in advanced ACS, which becomes obvious only at a point where the window of time for effective treatment (decompressive fasciotomy) has passed. Episodic intracompartmental pressure measurements can be performed using intra‐compartmental sensors (e.g., C2DX, Inc.)^[^ ^5^, ^6^, ^7^ ^]^ to aid decision‐making, but pressure alone is not determinative. While recent advancements in continuous intracompartmental pressure (ICP) monitoring devices (e.g., MYO1)^[^ ^8^, ^9^ ^]^ provide real‐time trends and improve diagnostic accuracy over episodic measurements, incorporating multimodal monitoring may further enhance the detection and management of ACS by addressing the limitations of relying solely on pressure. Moreover, current non‐invasive methods—such as Near‐infrared Spectroscopy (NIRS), tissue hardness measurements, Strain Elastography (SE), Shear Wave Elastography (SWE), and Pulsed Phase‐Locked Loop (PPLL) Ultrasound—are limited by shallow penetration, low specificity, and poor repeatability, making them inadequate for continuous, real‐time monitoring.^[^ ^3^ ^]^As a result of the high uncertainty associated with diagnosing ACS, patients are at risk for instances of missed diagnosis resulting in limb loss, and also conversely, from disfiguring surgery when ACS was not truly present.

To address these limitations, we introduce a multimodal sensor platform engineered with soft materials, enabling comprehensive and dynamic monitoring of ACS‐related pathophysiological changes. Our approach improves tissue conformity, reduces invasiveness, and enhances diagnostic accuracy compared to conventional diagnostic tools. The thin, flexible probe integrates sensors for pressure, changes of tissue oxygen saturation (StO_2_), and local blood flow rate, moving beyond the limitations of pressure monitoring alone. By leveraging soft materials with mechanical compliance, and thermal influences within the sensors, this platform ensures the independent operation of each sensor, enabling a multimodal sensing approach. This platform allows for simultaneous monitoring of multiple secondary symptoms, such as decreased tissue oxygen saturation and reduced blood flow, indicative of vascular and neural damage caused by increased compartment pressure. This study is, however, currently limited to prototype and feasibility testing. Complete validation of its advantages requires clinical patient data. This device is designed as an adjunct to physical examination, particularly for patients at moderate or high risk of limb compartment syndrome, including those with tibia fractures in both the pre‐operative and post‐operative periods. The probe inserts through a small stab incision, like that used for larger vascular catheters, making it applicable for open and closed injuries. By providing a comprehensive assessment of compartment pressure and tissue perfusion, this advanced technology enables reliable indications of the necessity for fasciotomy, reducing the likelihood of unnecessary procedures (false positives) and missed diagnoses. As a result, patients monitored with multimodal sensors, compared to those using standalone invasive or non‐invasive sensors, are expected to receive more accurate and timely treatment, leading to improved clinical outcomes and fewer unnecessary surgical interventions.^[^ ^1^, ^3^, ^10^, ^11^, ^12^, ^13^, ^14^, ^15^ ^]^ In addition, routine use of these sensors in clinical practice will provide healthcare professionals a powerful base of data with which to make informed decisions, ultimately improving patient care and reducing the risks associated with delayed or inaccurate diagnosis.

The sensors introduced here combine three digital measurement modalities co‐located on a thin, narrow probe to facilitate insertion directly into the compartment: i) pressure levels using a diaphragm‐shaped membrane with piezoelectric characteristics, ii) tissue oxygen levels through optical methods based on absorption in a reflection mode geometry, and iii) blood flow rates through measurements of thermal transport. The design includes i) three independent sensor modules integrated into a single, miniaturized probe, ii) continuous data digitalization and transfer via wireless data transmission, and iii) a scheme for deployment and removal using a peel‐away sheath introducer (PASI) configuration. Engineering analysis and comprehensive bench‐testing span i) validation studies to establish accuracy against established reference sensors, ii) in‐vivo evaluations to examine correlations between the sensor outputs in a simulated compartment environment using a porcine model, and iii) short‐ and intermediate‐term reliability studies to ensure stable operation over several hours in the porcine model.

## 2 Results and Discussion

### 2.1 Multimodal Sensing For Compartment Syndrome

**Figure 1:**
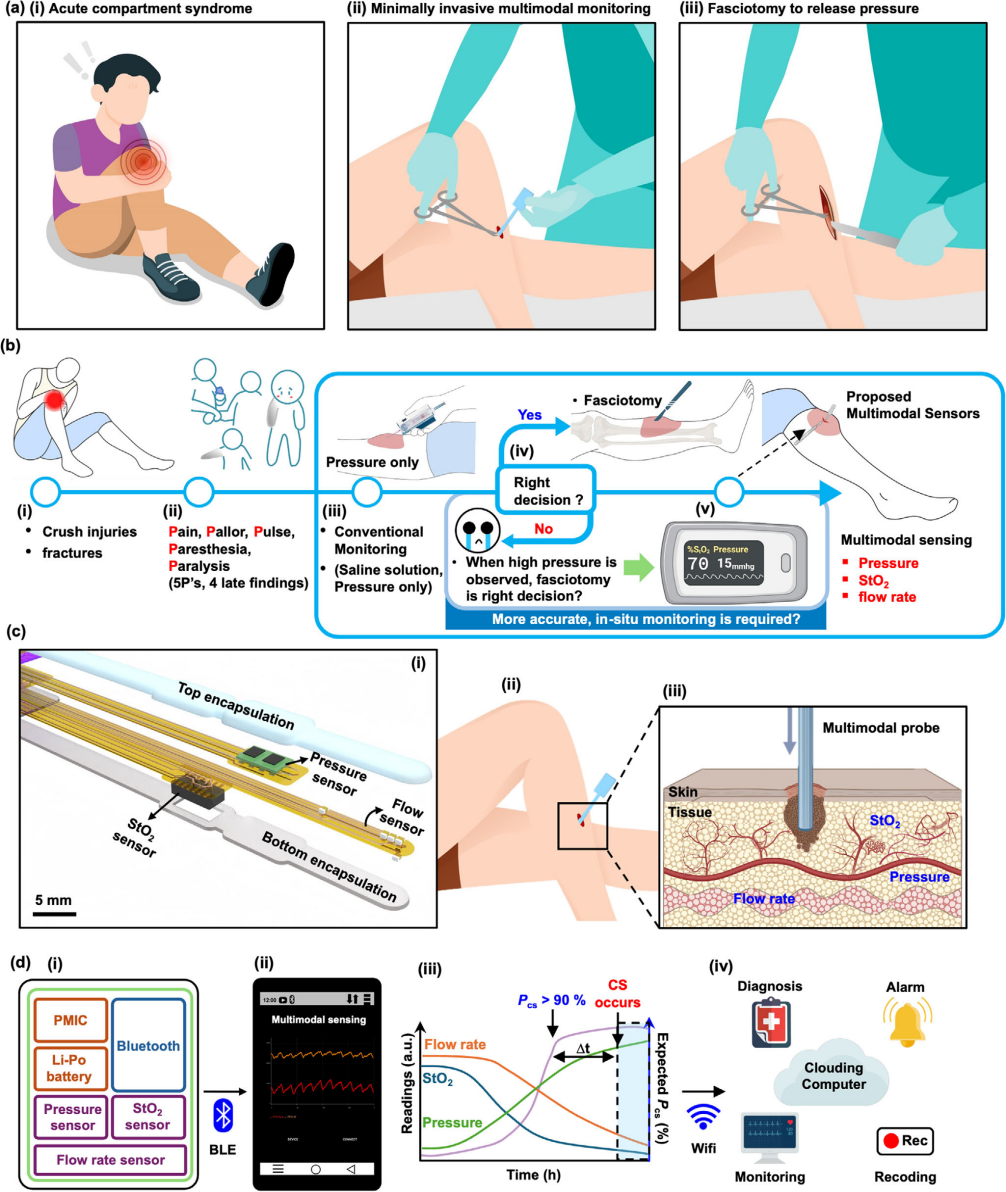
Multimodal sensing for compartment syndrome. a) Conceptual schematic illustration of events related to pressure injuries, the use of a multimodal sensor probe to monitor compartment syndrome, and fasciotomy. b) A comparison between conventional pressure monitoring and the multimodal sensor system for detecting compartment syndrome. The multimodal approach integrates sensors for pressure, StO_2_, and flow rate, allowing for improved clinical decision‐making. c) i) Exploded view schematic illustration of a multimodal sensor probe comprising a pressure sensor, an StO_2_ sensor, and a flow rate sensor, and ii, iii) cross‐sectional magnified views of the placement of these sensors within a leg compartment. d) i) Multimodal sensing of pressure, StO_2_, and flow rate, ii) Real‐time monitoring via a mobile device with Bluetooth connectivity, iii) Decision‐making assistance through binary convolutional neural network (BNN) analysis, and iv) conceptual schematic illustration of a digital healthcare system linked to cloud computing infrastructure and machine learning techniques, for remote digital healthcare and informed care decisions.

**Figure** **1**a(i–iii) presents a conceptual schematic illustration of events related to compartment syndrome. When physical trauma occurs, as shown in Figure 1a(i), a patient may develop injuries that result in excessive swelling or bleeding and associated increases in intracompartmental volume beyond the ability of the investing fascia to accommodate. As mentioned previously, increased pressure within the compartment impedes blood flow, resulting in a lack of oxygen and nutrients to surrounding tissues, potentially leading to impaired function and tissue death. ACS necessitates fasciotomy to release the pressure by cutting open the fascia. Accurate and early (within a few hours) decisions for such emergency surgical procedures can prevent permanent tissue damage. The multimodal sensor probes introduced here Figure 1a(ii) yield data that can effectively reduce the occurrence of both false negatives and false positives in diagnosis. As internal pressure increases within the compartment, a fasciotomy—a surgical procedure that involves cutting of the fascia to relieve tension—can alleviate the pressure and prevent ischemia (Figure 1a(iii)).

Figure 1b(i) shows the case of patients with crush injuries or fractures. These events lead to pain, pallor, pulselessness, paresthesia, and paralysis, as shown in Figure 1b(ii). Intra‐compartment pressure monitors (e.g., C2DX, Inc.) (Figure 1b(iii)) provide data to help physicians decide on the need for fasciotomy. When the diagnostic pressure differential (ΔP)—the difference between diastolic blood pressure (DBP) and ICP within an osteofascial compartment—remains below 30 mmHg for over 2 h, fasciotomy is strongly advised.^[^ ^1^ ^]^ Continuous monitoring and application of this threshold condition offers high diagnostic performance, with an estimated sensitivity of 94% and specificity of 98%.^[^ ^7^ ^]^ Pressure alone, however, may not capture the full clinical picture in all cases. Other sensors can confirm the diagnosis, detect early signs of complications, and/or offer insights in cases where pressure readings are ambiguous. Our multimodal sensor probe enables simultaneous monitoring of secondary symptoms—decreased tissue oxygen saturation and reduced blood flow, holding promise for enhancing physiological range prediction and providing a more comprehensive diagnostic tool for ACS. Although precise threshold values for these parameters are yet to be clinically established, the observed trends align with known physiological responses to pressure‐induced ischemia, suggesting their potential utility in differentiating disease severity and progression. Thus, a multimodal approach ensures a comprehensive and reliable assessment, reducing the risk of false positives or negatives that could arise from relying solely on pressure measurements. Furthermore, disadvantages of existing pressure sensors for this purpose follow from their sensitivity to orientation relative to gravity, the cumbersome, time‐consuming methods for implantation, and the need for continuous supervision by highly trained users. These shortcomings motivate the development of alternative methods for pressure sensing, and for capabilities to capture additional metrics associated with this condition, to enable timely and accurate decisions for fasciotomy. A key goal is to allow for early decision making with minimal risks of conducting unnecessary fasciotomy (false positive), as shown in Figure 1b(iv), where inappropriate surgery results in unnecessary physical damage, permanent scars, and wasted recovery time for the patient.

As a preferred approach, Figure 1b(v) illustrates a scenario in which a thin, flexible probe supports continuous, wireless monitoring of compartment syndrome with multiple sensor modalities, or an integrated sensor probe (Figure S1, Supporting Information). The probe shown here simultaneously detects three signals: i) internal pressure in the compartment, ii) StO_2_, and iii) flow rate relevant to blood circulation. Figure 1c(i) shows an exploded view schematic illustration of a multimodal sensor probe of this type. A thin, flexible printed circuit board (FPCB) with dimensions of 60 mm in length, 4 mm in width, and 1 mm in thickness serves as a supporting substrate, encapsulated in a soft silicone elastomer. As illustrated in Figure 1c(ii,iii), the probe inserts into a compartment through a small incision, with an external wireless control and communication interface that facilitates real‐time monitoring via a Bluetooth‐enabled smartphone application. Although optical sensors such as the one used for StO_2_ offer some penetration depth, implantation of the integrated sensor enables accurate tissue oxygenation measurement by minimizing signal degradation from superficial tissue layers and ensures spatial alignment among modalities without the need for external sensor positioning. Figure 1d illustrates the entire system: i) a setup capable of simultaneous detection of three signals (e.g., pressure, StO_2_, flow rate), powered by a Li‐Polymer battery, and equipped with a Bluetooth Low Energy (BLE) module; ii) a graphical display of measurement results for real‐time monitoring via a mobile device with Bluetooth connectivity (Figure S2, Supporting Information); iii) a binary convolutional neural network (BNN) analysis approach to assist in decision‐making; and iv) a conceptual cartoon of a digital healthcare system linked to cloud computing infrastructure and machine learning techniques, for remote digital healthcare and informed care decisions. In the context of an expected probability model (P_CS_) for patients with compartment syndrome as shown in Figure 1d(iii), enhanced accuracy in P_CS_ estimation is anticipated through the use of advanced training algorithms and decision‐making support systems. Figure S3 (Supporting Information) provides additional details, including input features for Bayesian Neural Network (BNN) simulations (e.g., pressure, flow rate, and StO_2_ levels as a function of time, along with maximum and minimum values, first (gradient) and second (inflection point) derivatives, and integrals of these parameters), and discussions of applications involving cumulative data for training and validating a BNN model integrated with Long‐Short‐Term Memory (LSTM) to predict CS with improved accuracy.

### 2.2 Design Features and Device Characteristics

**Figure 2:**
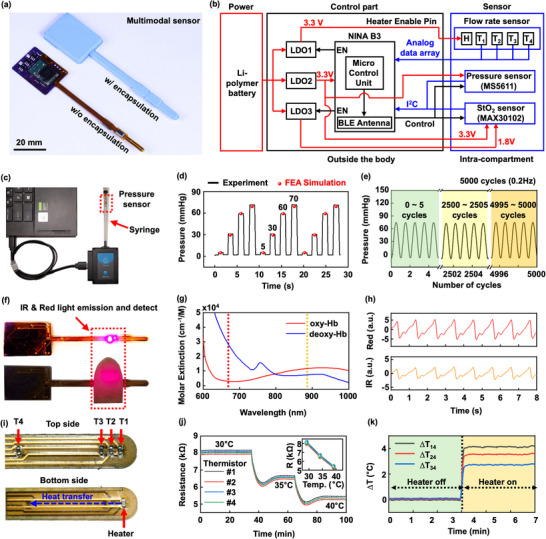
Design features and performance characteristics of multimodal sensor probes. a) Photograph of a device with and without a silicone elastomer encapsulation. b) Schematic diagram that shows modules for power, control, and sensing. c) Photograph of a pressure sensor and benchtop setup for characterization. d) Sensor response to pressure applied using a syringe, compared with FEA simulation. e) Repeatability of pressure response for 5000 cycles of application and release of pressure. f) Photograph of an StO_2_ sensor that relies on measurements of absorption of IR & red light emitted from integrated LEDs. g) Molar extinction of oxygenated and deoxygenated hemoglobin. h) Photoresponse at IR and red wavelengths, determined in a reflection mode measurement. i) Photograph of a flow rate sensor that includes four resistive temperature sensors (T_1_ – T_4_) and a resistive heater located behind T_1_. j) Resistance of the four temperature sensors as a function of temperature of the heater. k) Temperature change at each sensor during heater operation.

The probe involves modules for power, control, and sensing (**Figure** **2**a,b), with a biocompatible silicone elastomeric encapsulating structure (Ecoflex 00–30) as protection from biofluids and physical damage (Figure 2a). Figure 2b represents a circuit diagram of the system. The probe rests within the osteofascial compartment. The sensors quantify ICP, and changes of local blood flow and StO_2_ of the skeletal muscle within the target osteofascial compartment. A module for control and power supply resides outside the body, secured with Tegaderm (a transparent adhesive medical dressing; 3 M Inc.), to enable sensor operation, data collection and wireless transmission to a mobile device through BLE connectivity. The wireless design eliminates constraints associated with wired systems, which can restrict the patient or the medical staff in the dynamic clinical environment and lead to increased levels of noise and artifacts in the collected data. A battery powers the probe through three voltage regulators (LDO1 to LDO3). LDO2 provides a 3.3 V output that powers the main controller (NINA B3 chip), the StO_2_ sensor, and the pressure sensor. Although LDO1 also outputs 3.3 V, this power supports operation of the heater, thereby isolating it from the main voltage source (LDO2) to enhance power management. LDO3 supplies 1.8 V to the light‐emitting diodes (LEDs) in the StO_2_ sensor. To minimize standby power consumption, the heater of the flow sensor and the LEDs in the StO_2_ sensor activate only during continuous data monitoring and are turned off in standby mode. The pressure sensor and StO_2_ sensor communicate via I^2^C and transmit data to the main controller through two pins. With this configuration, the probe consumes ≈14 mA during continuous operation across all modalities (20 Hz, Figure S4, Supporting Information), a compact Li‐polymer battery (Capacity: 370 mAh, Length: 37 mm, Width: 25.5 mm, Thickness: 4.2 mm, Weight: ≈8 g) enables continuous monitoring for up to 24 h without requiring recharging or replacement. To further extend the operation time, the sampling rate can be reduced from 20 to 1 Hz, still sufficient for ACS monitoring. The resulting mode of operation consumes ≈4.2 mA (Figure S4, Supporting Information), enabling up to 88 h of continuous operation or allowing the use of a smaller battery with adequate operating life.

The pressure sensor employs a commercially available barometer (TE Connectivity, MS5611), modified to address requirements for the application considered here (Figure S5, Supporting Information). Specifically, the modification involves removing the metal cap to expose the diaphragm‐shaped membrane with piezo‐resistive components. After removing the cap, applying an encapsulating coating of a soft silicone elastomer directly on top of the active components ensures a uniform pressure transmission and isolates the active components from surrounding biofluids. This process effectively converts the barometer from a device designed to measure air pressure into one capable of accurately capturing mechanical pressures within the compartment. The results of finite element analysis (FEA) demonstrate that the soft elastomeric encapsulation effectively transmits the pressure to the surface of the sensor, ensuring a uniform distribution (Figure S6a, Supporting Information). Upon applying 70 mmHg of pressure to the outer surface of device, the pressure on the sensor membrane remains consistent, ranging from 69.8 to 70.5 mmHg. This result demonstrates the effectiveness of the design, as the sensor surface pressure closely aligns with the applied pressure (Figure S6b, Supporting Information) and shows a linear correlation (Figure S6c, Supporting Information). Validation includes hydrostatic pressure calibration in water (Figure S7, Supporting Information), FEA simulations to confirm effective pressure transmission through the elastomer (Figure S6, Supporting Information), and air pressure measurements in a syringe setup (Figure 2c). This system simulates pressures across a range relevant to those inside the compartment (Figure 2c). The results indicate reliable operation for both stepwise changes in pressure, with a response time of 0.01 to 0.1 s, closely matching the FEA predictions (Figure 2d). Additionally, the sensor demonstrates stable performance during dynamic variations over 5000 cycles within a range of 0 to 70 mmHg (Figure 2e). Built‐in temperature compensation ensures accurate measurements throughout a range relevant to body temperature (Figure S8, Supporting Information). As illustrated in Figure 2d, the response time is less than one second, which is adequate for pressure sensing in ACS monitoring, where rapid fluctuations are not typically expected.^[^ ^1^ ^]^

The StO_2_ sensor uses an integrated pulse oximetry module (MAX30102 by Maxim Integrated) that includes infrared (IR; 880 nm) and red (660 nm) LEDs, along with a photodetector (PD) that is responsive to wavelengths ranging from 600 to 900 nm (Figure 2f). The sensor detects light reflected from adjacent tissue, and provides photoplethysmography (PPG) data (Figure 2h), which is then processed to determine StO_2_ (%) (Figure 2h) using molar extinction features in oxygenated (*HbO*_2_) and deoxygenated hemoglobin (*Hb*) (Figure 2g).

(1) $$\mathrm{St}O_{2}\left(t\right)=\frac{\mathrm{Hb}O_{20}+\Delta \mathrm{Hb}O_{2}\left(t\right)}{\mathrm{Hb}O_{20}+\Delta \mathrm{Hb}O_{2}\left(t\right)+\;Hb_{0}+\Delta Hb_{0}\left(t\right)}$$

Details appear in the Experimental Section. In many cases, the data contain oscillatory components that can be used to determine pulse rate and pulse rate variability. The calculation of tissue oxygenation yields relative changes, rather than absolute values. This approach is consistent with the capabilities of the MAX30102 sensor, which measures PPG signals at two wavelengths (660 and 880 nm). We compared our results to validate the relative measurements with data obtained from a commercial system (Vioptix), as shown in Figure S9b (Supporting Information).

The flow rate sensor includes four thermistors (T_1_ – T_4_) on the top side and a resistive heater (H) on the bottom (Figure 2i). The thermistors record temperatures as changes in resistance, through a linear calibration (Figure 2j). Upon activation of the heater behind thermistor T_1_, heat transfers in the direction from T_1_ to T_4,_ in a distribution that can be quantified from the responses of these thermistors (Figure 2k). This distribution depends on the local thermal transport properties of the adjacent tissue, which are influenced by the heater and the overall rate of blood flow. Notably, the local tissue temperature only serves as an initial reference before the heater activation and does not affect the temperature difference (ΔT_14_) between thermistors (Figure S10, Supporting Information), and Figure S11 (Supporting Information) confirms negligible thermal interference to the StO_2_ sensor from the flow sensor. Previous reports provide information on calculating changes in flow rate, with details in the Experimental Section.^[^ ^16^ ^]^ Earlier studies validate the reliability of the sensor in monitoring trends in flow rate across complex vascular geometries. This study applies the sensor to ACS, focusing on detecting temporal changes in blood flow rather than precisely estimating absolute values, which is crucial for early diagnosis. Experimental validation confirms that the proposed device platform provides sufficient performance in pressure, tissue oxygenation, and flow rate sensing for continuous ACS monitoring.

### 2.3 Design Considerations for Optimized, Independent Operation of the Sensors

**Figure 3:**
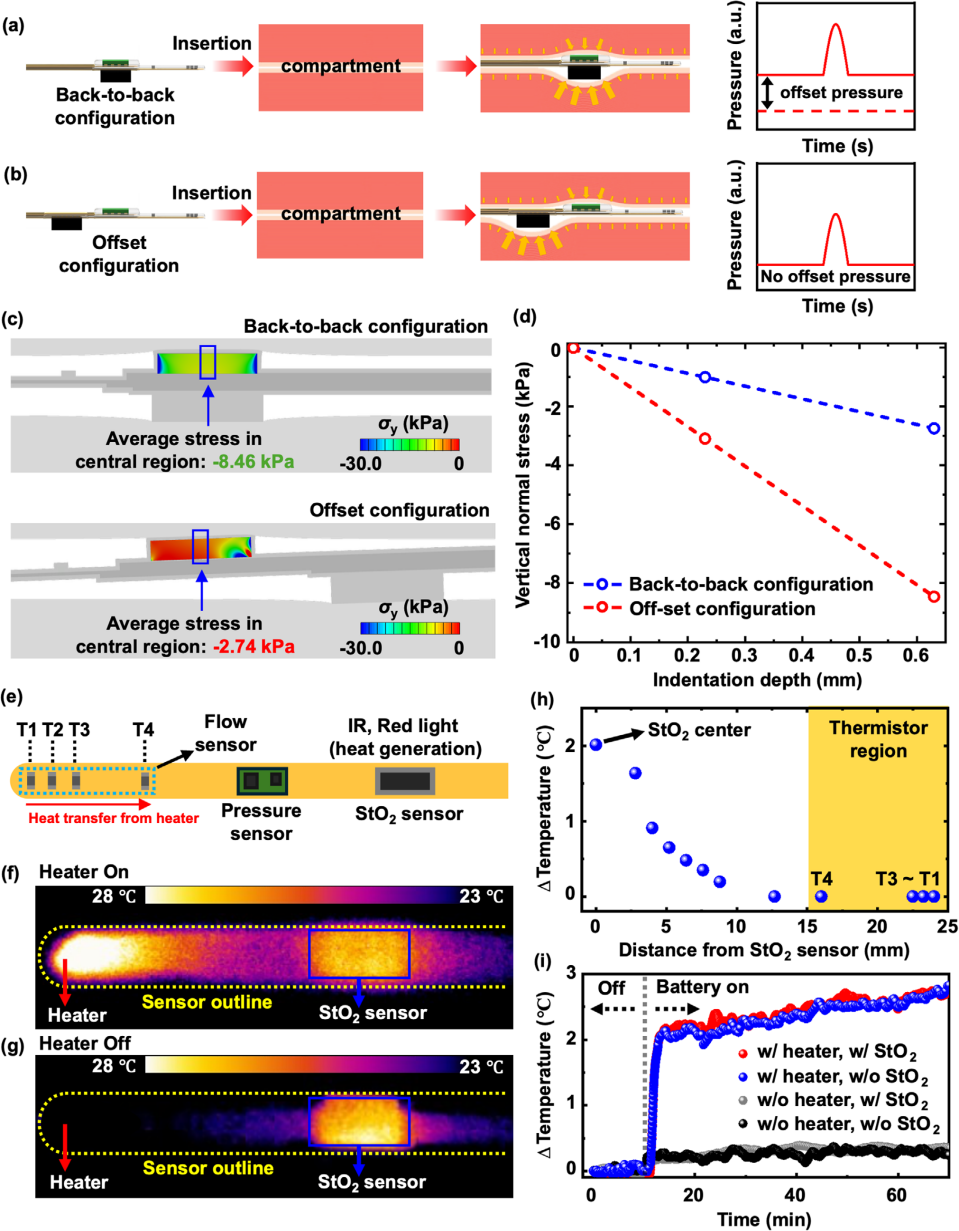
Design considerations for optimized, independent operation of the sensors. a) Schematic illustration of multimodal sensor probes with a) back‐to‐back and b) offset configurations. c) Computed distributions of stress for these two configurations upon insertion into a material with a Young's modulus of 166 kPa, comparable to that of most soft biological tissues. d) Plot of the shift in the pressure sensor versus total tissue compression depth for both back‐to‐back and offset configurations. e) Schematic illustration of the sensor locations and direction of heat transfer. f,g) Measured temperature distributions across the probe during operation of the heater and oximeter. h) Measured changes in temperature as a function of distance from the StO_2_ sensor during operation. i) Measured change in temperature of sensor T_4_ as a function of time during operation of the heater and the StO_2_ sensor.

Careful choices in layout enable the various sensors in the multimodal sensor probe to operate in an accurate and independent manner, even with slight spatial offsets within the same osteofascial compartment. Illustrations of two representative configurations highlight an example of the effect of mechanical coupling that must be minimized (**Figure** **3**a,b). During use, elastic recovery of the muscular tissue after insertion can lead to forces on the device. Without an offset between the pressure sensor and the StO_2_ sensor (back‐to‐back configuration), the relatively thick structure (≈3 mm) of the pressure sensor leads to significant mechanical stresses upon insertion into the tissue. These stresses can prevent accurate measurements of pressure. A configuration that separates the pressure sensor from the StO_2_ sensor by a certain distance along the probe (≈9 mm; offset configuration) reduces the thickness of the region of the pressure sensor to 1 mm, thereby minimizing the development of stresses in this region. FFEA results show the shifts in pressure response that result for these two configurations (Figure 3c,d). In both cases, the shift depends linearly on the extent of tissue compression. A value of 630 µm serves as the indentation depth, defined as the difference between the total thickness of the sensors (3.63 mm) and the arbitrarily set compartment gap (3 mm). This value follows from an assumed expansion (≈20%) of the muscular compartment to accommodate the multimodal sensor. The offset configuration reduces the shift by ≈60% compared to the back‐to‐back configuration (e.g., 2.74 kPa vs 8.46 kPa).

The flow rate sensor relies on changes in effective thermal transport properties that result from flow. As such, the operation can depend on thermal interference from adjacent sensors (Figure 3e), specifically that for StO_2_, which involves LEDs that produce not only light but also heat. Figure 3f shows the temperature distribution measured by an infrared camera during simultaneous operation of the flow and StO_2_ sensors. The changes in temperature induced by the StO_2_ sensor alone are confined, however, to a localized area (Figure 3g). The peak change in temperature due to heating from the StO_2_ sensor is ≈2 °C, with negligible change at distances larger than ≈12.5 mm along the length of the probe (Figure 3h). Thus, thermal interference can be avoided by locating the thermistors (T_1_ – T_4_) at distances of 16, 22.5, 23.25, and 24 mm, respectively, from the StO_2_ sensor. Figure 3i presents time‐dependent changes in temperature for thermistor T_4_, nearest to the StO_2_ sensor for operation i) with heater and with StO_2_ sensor, ii) with heater and without StO_2_ sensor, iii) without heater and with StO_2_ sensor and iv) without heater and without StO_2_ sensor. The results show responses that are independent of the operation of the StO_2_ sensor.

### 2.4 Strategies for Minimally Invasive Implantation

**Figure 4:**
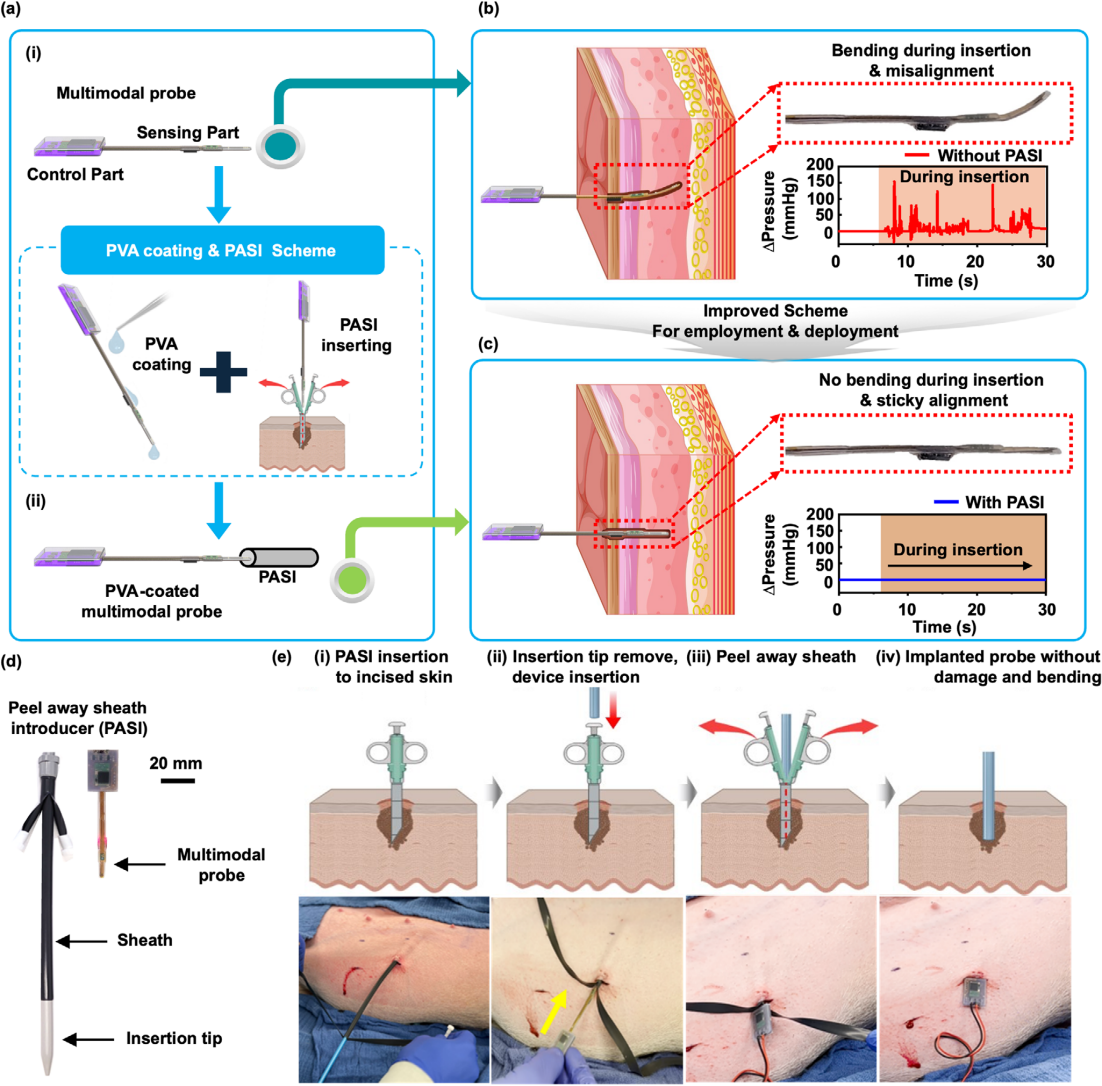
Strategies for minimally invasive deployment. a) Schematic illustrations of two scenarios: i) direct insertion of the multimodal sensor probe without additional encapsulation, and ii) insertion of the probe with an overcoat of PVA, with the PASI method. b) Schematic and photographic representations of the multimodal sensor probe without PASI, highlighting the external pressure applied during insertion. c) Corresponding schematics and photographs of the probe with PASI, showing the application of external pressure during deployment. d) Optical images of the PASI components—the sheath and insertion tip—alongside the multimodal sensor probes. e) Step‐by‐step procedure for minimally invasive deployment of the multimodal sensor probe. i) Forming a skin incision to facilitate insertion. ii) Inserting the probe using PASI, iii) Peeling away the sheath, iv) Positioning the multimodal sensor probe within the targeted compartment.

**Figure** **4** illustrates the process of deploying the device in a minimally invasive manner, comparing two different approaches and detailing the procedural steps involved. Figure 4a highlights schematic diagrams of these two insertion techniques. The first method i) involves direct insertion without any additional encapsulation, using only Ecoflex 00–30 for protection from biofluids. This method, while straightforward, exposes the probe to mechanical stresses and interference from surrounding tissues. The second scenario ii) involves an overcoat of polyvinyl alcohol (PVA) and a PASI. The PVA coating not only acts as a protective layer but also increases the bending stiffness (Bare device: 0.03 N mm^−1^ to 0.9 mm of PVA coating: 0.08 N mm^−1^) of the probes during insertion (Figure S12, Supporting Information). This added stiffness facilitates navigation through tissue. The PASI method ensures controlled and precise positioning of the probe within the tissue, as the PVA layer begins to resorb once in the tissue. Prior to resorption, the PVA aids in anchoring the position of the device in the tissue. Specifically, the PVA‐coated device requires four times more force to be pulled out of the tissue after insertion compared to the bare and encapsulated probes (Figure S13, Supporting Information). Figure 4b provides both schematic illustrations and photographic representations of the probe when inserted without the PASI. The illustration emphasizes the external pressure that must be applied during this process, which can be inconsistent and may require significant manual effort from the surgeon. The accompanying photograph further depicts the practical challenges associated with this method, including potential deformation or misalignment of the probe. By contrast, Figure 4c illustrates the use of the PASI for controlled insertion of the probe insertion. This method reduces the risk of damage to both the probe and surrounding tissues, resulting in a more reliable and accurate placement.

Figure 4d illustrates the components of the PASI. The optical images illustrate the key elements—the sheath and the insertion tip—that are essential for the successful application of this method. The sheath serves as a protective barrier, while the insertion tip guides the probe to its intended location within the body. Figure 4e shows step‐by‐step deployment procedure. The process begins with a small incision in the skin to facilitate the entry of the probe (i). Next, the probe inserts into the tissue with the PASI (ii). Once the probe is in place, the sheath safely peels away (iii), leaving the multimodal sensor probe positioned within the targeted compartment (iv). Photographs of this process appears at the bottom panel. In addition, the probe, encapsulated in a soft silicone elastomer and inserted via a controlled PASI mechanism, maintains mechanical and functional stability after retrieval and re‐implantation.

### 2.5 Multimodal Sensing For Compartment Syndrome in a Porcine Model

**Figure 5:**
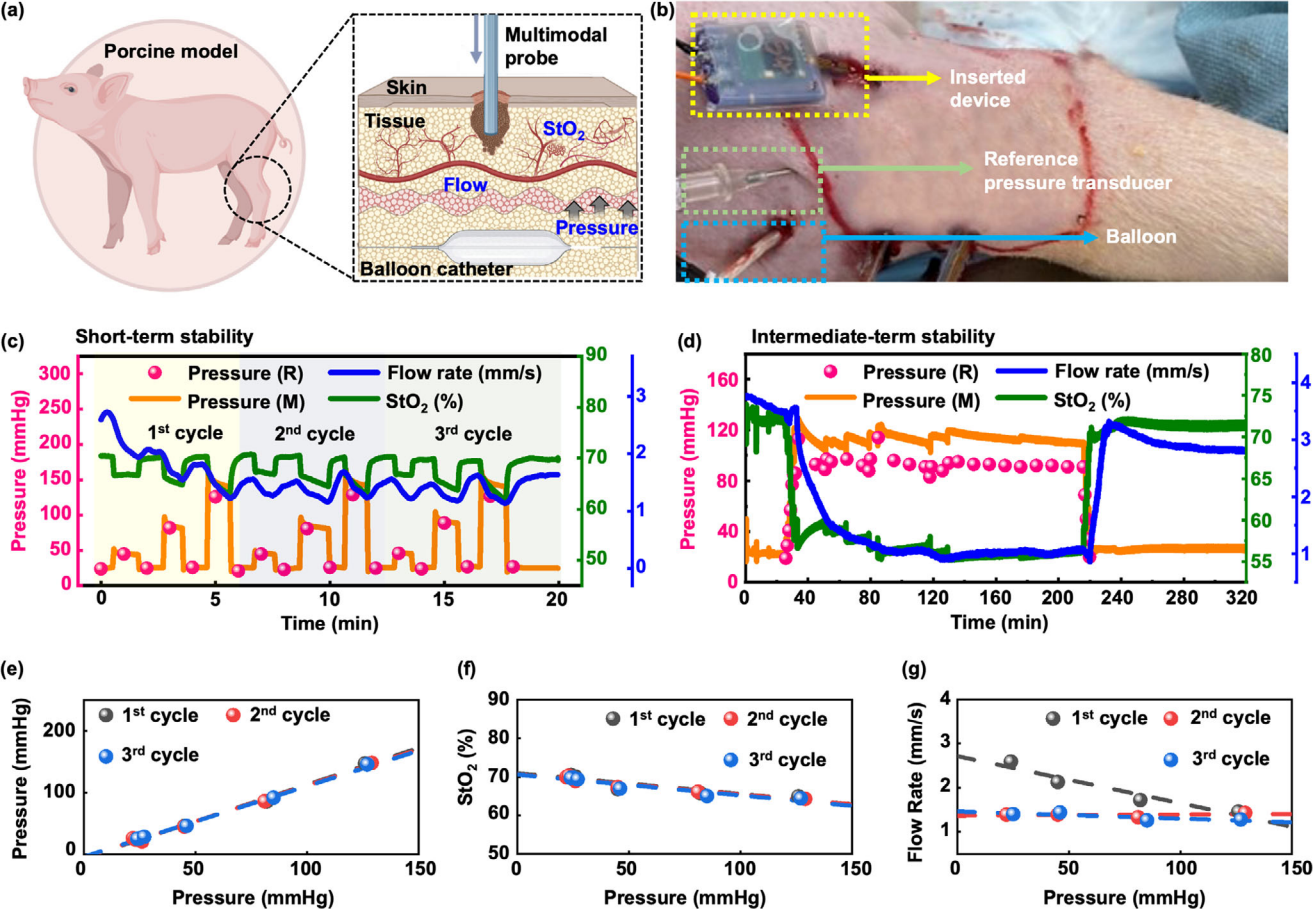
Multimodal sensing in a porcine model. a) Schematic illustrations of the setup to validate the probe in a porcine model. b) Photograph after insertion of the probe, balloon catheter, and reference pressure sensor. c) Multimodal sensing during three cycles of increasing and decreasing the pressure by inflating and deflating the balloon, respectively. d) Multimodal sensing during an extended period (≈3 h) of pressure loading. Responses and correlations of e) pressure, f) StO_2_, and g) flow rate to the reference pressure loading in (c).

In‐vivo experiments utilizing a porcine model demonstrate the effectiveness of these multimodal sensors for real‐time monitoring of critical physiological parameters—blood flow, StO_2_, and pressure—during a simulation of internal leg compartment syndrome (**Figure** **5**a). This experiment mimics the conditions of compartment syndrome, where increased internal pressure within a osteofascial compartment leads to reduced blood flow and oxygen delivery, potentially resulting in tissue damage. Figure 5a includes a schematic representation of the experimental setup and a photograph of the procedure (Figure 5b). The simulation follows a general protocol for modeling ACS by using stepwise inflation of an intercompartmental submuscular pretibial balloon catheter placed near the multimodal sensor probe, enabling controlled adjustments of internal compartment pressure.^[^ ^17^, ^18^, ^19^ ^]^ This process increases the pressure incrementally from 0 to 50, 100, and 150 mmHg over the course of one minute, while the probe continuously records data. Preliminary feasibility studies were conducted prior to the planned detailed experiments. The pressure and StO_2_ data measured by the multimodal sensor probe in the porcine tissue model aligned with the data obtained from reference sensors, confirming the reliable sensing capabilities of the probe even after implantation (Figure S9, Supporting Information).

As depicted in Figure 5c, the pressure measurements from the multimodal sensor probe closely follow the trends observed with the reference pressure transducer, thereby validating the accuracy of the probe. Notably, the data reveals inverse relationships between pressure and both flow rate and StO_2_ levels. As the compartment pressure rises, blood flow decreases, leading to reduced oxygen delivery—an expected outcome consistent with the pathophysiology of compartment syndrome. The results demonstrate stable sensing and repeatability in the response over these time periods. The initial cycle of an increase in pressure leads to a gradual decrease in flow rate, likely affected by the process of thermal equilibration of the probe with the surrounding tissue (Figure S14, Supporting Information).

The results of intermediate‐term stability tests appear in Figure 5d. Stability over these timescales, specifically for more than 2 h, is essential for the accurate determination of compartment syndrome. Figure 5d illustrates that the ICP response (denoted as pressure (M)), measured continuously at a sampling rate of 20 Hz closely aligns with the trend of the reference data (denoted as pressure (R), Pressure transducer), which was recorded at 10 min intervals. The multimodal sensor probe provides a stable measurement of the three parameters over a 3 h period of pressure loading. Furthermore, the flow rate and StO_2_ levels correspond logically to changes in ICP within the compartment during total ≈5 h of experiment. Notably, the changes in flow rate consistently occur at a delayed time relative to those in ICP and StO_2_ levels. This delay likely follows from the timescales for temperature equilibration within the tissue. Figure 5e–g presents quantitative evaluations of the measured pressure, StO_2_, and flow rate in response to induced pressure loading, derived from Figure 5c. The data demonstrates i) consistent maintenance of pressure, StO_2_, and flow rate values over three cycles of dynamic short‐term pressure loading, and ii) a linear relationship between the reference pressure (x‐axis) and each parameter measured by the multimodal sensor probe (y‐axis). These results confirm reliable and reproducible sensing capabilities.

The consistency and reliability of the multimodal sensor probe in capturing real‐time data on pressure, blood flow, and StO_2_ during compartment syndrome simulation underscore its potential clinical utility. The inverse relationship between pressure and both flow rate and oxygen saturation provides important insights into the early detection and management of compartment syndrome, where timely intervention is critical to prevent irreversible tissue damage. In addition, biocompatible encapsulation and its successful passage of standardized biological safety tests (Figures S15–S22, Supporting Information) support its suitability for implantation in clinical scenarios.

## 3 Conclusion

This paper introduces a multimodal sensor platform for early diagnosis of ACS that supports simultaneous measurements of pressure, StO_2_, and blood flow in the form of a thin, flexible, and wireless probe. This system offers real‐time, multimodal data with continuous monitoring capabilities, contributing to the detection of compartment physiology, by simultaneously tracking secondary symptoms—decreased tissue oxygen saturation and reduced blood flow, both indicative of vascular and neural damage. The platform improves upon conventional diagnostic methods by providing multimodal data that could enhance the sensitivity and specificity of ACS detection, offering complementary insights through ICP, blood flow, and relative StO_2_ to support timely interventions like fasciotomy or identify issues such as probe malfunction or displacement. This advance is enabled by the multimodal sensing of secondary symptoms, which not only provides a more comprehensive physiological context, but also significantly reduces the likelihood of misinterpretation caused by muscle contractions or external pressure. The PASI scheme allows for minimally invasive insertion, enhancing the ease of deployment and sensor placement. The modular design, coupled with wireless data transmission, supports 24 h operation without recharging, as a practical tool for dynamic clinical environments. Our approach leverages scalable manufacturing techniques and integrates commercially available components, to increase the potential for clinical translation and widespread adoption. This platform complements existing methods focused solely on pressure measurements and introduces additional opportunities for enhanced diagnostic precision through multimodal sensing. While this study demonstrates the feasibility of multimodal integration, further validation is necessary to quantify the benefits of additional modalities, particularly in reducing false‐positives and false‐negatives, or detecting early signs of ACS compared to pressure‐only systems. Future research will focus on refining the platform, including clinical trials of 24–48 h involving patients at risk for ACS. These trials will directly compare the diagnostic accuracy of multimodal sensing to continuous single‐modality ICP monitoring and evaluate the capability of these systems to provide earlier detection.

Additionally, while the current approach calculates relative changes in StO_2_ rather than absolute values, this study contributes to the ongoing effort to validate and standardize relative measurements against established baselines using commercial systems (e.g., Vioptix). Robust verification and validation procedures will be essential for future work to enable accurate absolute calibration of StO_2_.

In addition to monitoring pressure, flow rate, and StO_2_, approaches that incorporate real‐time measurements of metabolites, such as lactate and K⁺, may provide additional insights into muscle and vascular health. The application of machine learning algorithms may also enhance decision‐making based on real‐time data streams. This technology holds significant promise for advancing clinical management of ACS and improving patient outcomes by providing early and reliable diagnoses, ultimately contributing to broader applications in remote monitoring, digital healthcare, and emergency medical interventions.

## 4 Experimental Section

### Fabrication of Multimodal Sensor Probe

Lead‐free solder paste (Sn/Bi/Ag) formed electrical connections between each surface‐mounted component—including commercial resistors, capacitors, a linear voltage regulator, the nRF52840 Bluetooth module, thermistors, an oximeter, and a pressure sensor—and the flexible printed circuit board (FPCB, PCBway). The multimodal sensors comprise three FPCBs, each ≈3 mm wide and 62 mm long, for independent measurements of ICP, flow rate, and StO_2_ levels (Figure S23a, Supporting Information). Additionally, a rigid PCB located outside the body serves as the main controller for Bluetooth communication and power supply. The pressure sensor and StO_2_ sensor rely on modified version of commercially available components: a barometer (MS5611) for pressure sensing and an oximeter (MAX30102) for StO_2_ measurement. The flow sensor consists of four negative temperature coefficient (NTC) thermistors (B = 3380 K, 10 kΩ) and one heater (1 kΩ). In the FPCBs, a thin polyimide layer (100 µm) served as the substrate, and Cu lines (18 µm) functioned as the interconnects. The assembly process began with mounting the three sensors on their respective FPCBs, while the wireless module and power management unit (PMU) were mounted on the rigid PCB. After the fabrication of the three sensors and the main body, assembling into a single device relied on a soldering process (Figure S23b, Supporting Information). The pressure and flow sensors were positioned on the top side of the FPCBs, with the StO_2_ sensor on the bottom side. Spacing the three sensors apart by a specific distance minimized interference. The top and bottom encapsulation layers were fabricated using a pair of molds, designed with computer‐aided design software (SOLIDWORKS 2019, Dassault Systems) and machined from aluminum using a milling machine (MDX‐540, Roland DGA). Ecoflex 00–30 was cast onto these molds and cured at 75 °C for 60 min to form the encapsulating structures. Biocompatible encapsulation layer (Ecoflex 00–30) applied to the entire device, including the implantable probe and the main body, protected against physical damage and prevented electrical malfunctions caused by biofluid penetration. After fabrication, operational tests of the pressure and flow sensors verified proper function (Figures S7 and S24, Supporting Information).

### Calculation of Flow Rate

The following equation determined the rate of blood flow from measured parameters:

(2) $$\Delta T\approx \frac{qR/\lambda }{1+0.76s\sqrt{\frac{uR}{\alpha_{\mathrm{fluid}}}}}F\left(\frac{r}{R}\right)$$

where *ΔT* is the change in temperature for each thermistor, 𝛼_fluid_ is the thermal diffusivity of the fluid (blood)*, λ* is the effective thermal conductivity of the tissue, *q* is the heat flux of the heater, *u* is the flow velocity *r* is the distance between each thermistor and the heater, *R* is the radius of the heat spreader, *s* is the blood content, and $F\;\left(\frac{r}{R}\right)=\int_{0}^{\infty }b_{0}\;\left(\gamma r\right)\;b_{1}\left(\gamma R\right)\frac{d\gamma }{\gamma }$ , *b*_0_ and *b*_1_ are Bessel functions of the first kind for orders 0 and 1, respectively. Details appear elsewhere.^[^ ^16^ ^]^

### Calculation of StO_2_ Level

The value of StO_2_ can be determined from photoplethysmography (PPG) signals at red and IR wavelengths obtained by the MAX30102, with calculations performed subsequently. Optical densities as a function of time *OD*(λ, *t*) can be expressed as:

(3) $$OD\left(\lambda ,t\right)=-\ln\left(\frac{I_{t}\left(\lambda \right)}{I_{0}\left(\lambda \right)}\right)$$

where *I_t_* (λ) and *I*_0_(λ) are the intensities at a time *t* and an initial value, respectively, computed for two wavelengths (IR and red in this case). Generally, the PPG signal contains an alternating current (AC) component (pulsatile signal from cardiac cycles) and a direct current (DC) component (offset value). Passing the signal through a 0.1 Hz low pass filter removes the AC component, to allow calculation of the changes of the oxygenated hemoglobin (Δ[*HbO*_2_](*t*)) and deoxygenated hemoglobin (Δ[*Hb*](*t*)) as a function of time according to the Lambert‐Beer law for diffusive media:

(4) $$\begin{array}{cc} \left[\begin{array}{c} \Delta \left[\mathrm{Hb}O_{2}\right]\left(t\right)\\ \Delta \left[\mathrm{Hb}\right]\left(t\right) \end{array}\right] & =\frac{1}{q}{\left[\begin{array}{cc} \varepsilon_{\mathrm{Hb}O_{2}}\left(\lambda_{1}\right)\cdot \mathrm{DPF}\left(\lambda_{1}\right) & \varepsilon_{\mathrm{Hb}}\left(\lambda_{1}\right)\cdot \mathrm{DPF}\left(\lambda_{1}\right)\\ \varepsilon_{\mathrm{Hb}O_{2}}\left(\lambda_{2}\right)\cdot \mathrm{DPF}\left(\lambda_{2}\right) & \varepsilon_{\mathrm{Hb}}\left(\lambda_{2}\right)\cdot \mathrm{DPF}\left(\lambda_{2}\right) \end{array}\right]}^{-1}\\ & \;\times \left[\begin{array}{c} \mathrm{OD}\left(\lambda_{1},t\right)\\ \mathrm{OD}\left(\lambda_{2},t\right) \end{array}\right] \end{array}$$

where *q* is the distance between LED and PD, and *DPF(*λ) is the differential path distance factor at the wavelength of interest. $\varepsilon_{\mathrm{Hb}O_{2}}$ and ε_Hb_ are molar extinction coefficients of oxygenated hemoglobin and deoxygenated hemoglobin, respectively. The value of StO_2_ follows from:

(5) $$\mathrm{St}O_{2}\left(t\right)=\frac{\mathrm{Hb}O_{20}+\Delta \mathrm{Hb}O_{2}\left(t\right)}{\mathrm{Hb}O_{20}+\Delta \mathrm{Hb}O_{2}\left(t\right)+Hb_{0}+\Delta Hb_{0}\left(t\right)}$$

where *HbO*_20_ and *Hb*_0_ are the amount of oxygenated hemoglobin and deoxygenated hemoglobin at initial state. Both parameters are approximated by the ratio of the concentration and molar mass of hemoglobin ($HbO_{20}+H\;b_{0}=\frac{Concentration_{\mathrm{Hb}}}{Molar\;Mass_{\mathrm{Hb}}}\;$) and the StO_2_ in the tissue under normal conditions.

### In Vitro Evaluation of Sensor Performance

Benchtop tests with a syringe and water column verified the reliability of the pressure sensor. A syringe pump applied 5000 pressure cycles, and 2 additional cycles were performed using a water column to confirm zero hysteresis and ensure reproducibility. Thermistor calibration was carried out by recording resistance values in an environmental chamber across a temperature range of 30 – 40 °C. Temperature distributions generated by the heater and StO_2_ sensor in the multimodal sensor probe were captured using an infrared camera (FLIR A600, Teledyne FLIR LLC) under four scenarios: i) both heater and StO_2_ on, ii) heater on and StO_2_ off, iii) heater off and StO_2_ on, and iv) both heater and StO_2_ off. The extraction force of implanted multimodal sensor probes, with or without a PVA layer, was measured using a tensile tester (Mark‐10 ESM303) equipped with a 10 N force gauge (Mark‐10 Corporation). For minimally invasive deployment, a Peel‐Away Sheath Introducer (16 Fr, Cook Medical Inc.) was used to insert the multimodal sensor probe into the tissue.

### Probe Implementation Protocol

The entire probe head is positioned within the intracompartmental muscle and is inserted using a peel‐away sheath with a trocar, which is introduced through a stab incision made with a 15‐blade scalpel. The procedure is conducted under local anesthesia to minimize discomfort. The precise placement within the muscle (central, peripheral, proximal, or distal regions) is not critical, as the device does not depend on a fluid column for pressure measurement. Similarly, insertion angle and depth are not significant factors affecting the sensor's functionality.

The probe is particularly well‐suited for subcutaneous compartments, such as the anterior compartment of the leg or the extensor compartment of the forearm. For deeper compartments, such as the deep posterior compartment of the leg, ultrasound guidance will be utilized to ensure accurate and safe placement, particularly in cases where palpation alone is insufficient for proper positioning.

### In Vivo Experiments using Porcine Compartment Model

The study was approved by the Institutional Animal Care and Use Committee (IACUC) at Washington University School of Medicine under protocol 21–0145. All animal procedures were conducted in accordance with established guidelines. A total of 4 domestic swine (100–110 lbs, sourced from Oak Hill Genetics) were used for the experiments. Preliminary feasibility tests in Figure S9 (Supporting Information) were conducted in two animals, and each in vivo experiment in Figure 5c,d was conducted in the other two animals. The animals were initially sedated and positioned dorsally, with deep anesthesia induced and maintained by the Division of Comparative Medicine (DCM) staff. Vital signs, including rectal and peripheral temperature, oxygen saturation, and heart rate, were continuously monitored throughout the procedure. The study focused on the anterior compartments of both hind limbs. A #8 French plastic trocar was inserted through the skin at the medial distal tibia and carefully advanced between the anterolateral surface of the tibia and the deep fascia of the anterior osteofascial compartment, exiting laterally at the tibial head. Once a balloon catheter was positioned within the osteofascial compartment, the trocar was removed. ACS was induced by inflating the balloon catheter with saline, increasing ICP to 30 mmHg above the mean arterial pressure (MAP), which restricted blood flow to the muscle and simulated ACS. Throughout the procedure, all physiological parameters were continuously monitored using attached sensors. Upon completion of the experiments, the animals were humanely euthanized with pentobarbital.

### Finite Element Analysis

The commercial software package ABAQUS (version 2022) was used to perform FEA on the device, which was modeled using four‐node 3D stress elements (C3D4H). During assembly, the mass centers of the devices were precisely aligned with the center of the coordinate system. The top and bottom encapsulations were bonded using a “tie” constraint, while the sensors and FPCBs were embedded within the encapsulations using an “embedded region” constraint. An “encastre” boundary condition was applied at one end of the device to ensure stability. A refined mesh, with feature sizes smaller than 10% of the probe's width, was employed to enhance accuracy. During the simulation, pressures of 5, 30, 60, and 70 mmHg were applied to the outer surface of the probe. The mechanical properties of Ecoflex 00–30 were captured using a second‐order, incompressible Yeoh hyper‐elastic constitutive model, with an elastic modulus (E) of *E*_Ecoflex_ = 60 kPa (the relevant parameters being *C*_10_ = 10 kPa, *C*_20_ = 1 kPa). PI, chips, and FR‐4 were modeled as linear elastic materials with the following properties: *E*_PI_ = 2.5 GPa, ν_PI_ = 0.34 for PI; *E*_chips_ = 170 GPa, ν_chips_ = 0.28 for chips; *E*_FR4_ = 10 GPa, ν_FR4_ = 0.25 for FR‐4.

### Statistical Analysis

Device validations were independently performed using separate devices and repeated with consistent results. Representative data are presented in Figures 2 and 3. For Figure 5c,d, each test was conducted using one porcine model.

## Conflict of Interest

The authors declare no conflict of interest.

## Supporting information



Supporting Information

## Data Availability

The data that support the findings of this study are available from the corresponding author upon reasonable request.
