# Supplementary material for: Minimally Invasive, Bioadaptive Multimodal Sensor Probe with Safe Deployment for Real‐Time Acute Compartment Syndrome Diagnosis
Source: Adv Sci (Weinh). 2025 Jul 17;12(33):e06942. doi: 10.1002/advs.202506942 (PMC12412494; doi:10.1002/advs.202506942)
Supplement: Supplementary file 1 — Supporting Information [file ADVS-12-e06942-s001.docx]

Supporting Information

**Minimally Invasive, Bioadaptive Multimodal Sensor Probe with Safe Deployment for Real-Time Acute Compartment Syndrome Diagnosis**

*Seung Gi Seo, Seungyeob Kim, Seonggwang Yoo, Seyong Oh, Haiwen Luan, Zengyao Lv, Bosung Kim, Shupeng Li, Di Lu, Jong Uk Kim, Yaeshin Park, Jae Hee Lee, Hyeon-Bin Jo, Amanda M. Westman, William Moritz, Joseph Ribaudo, Yonggang Huang, Mitchell A. Pet*, Sung Hun Jin*, John A. Rogers**


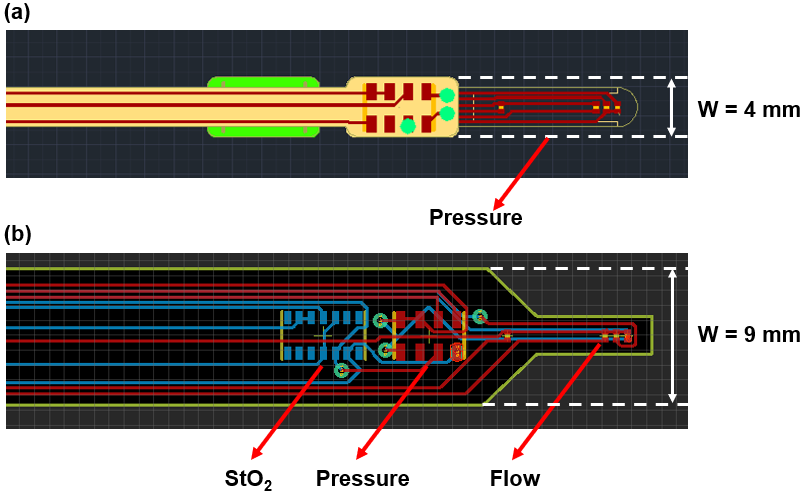


**Figure S1.** Circuit design of multimodal probe integrated into a single PCB.

**
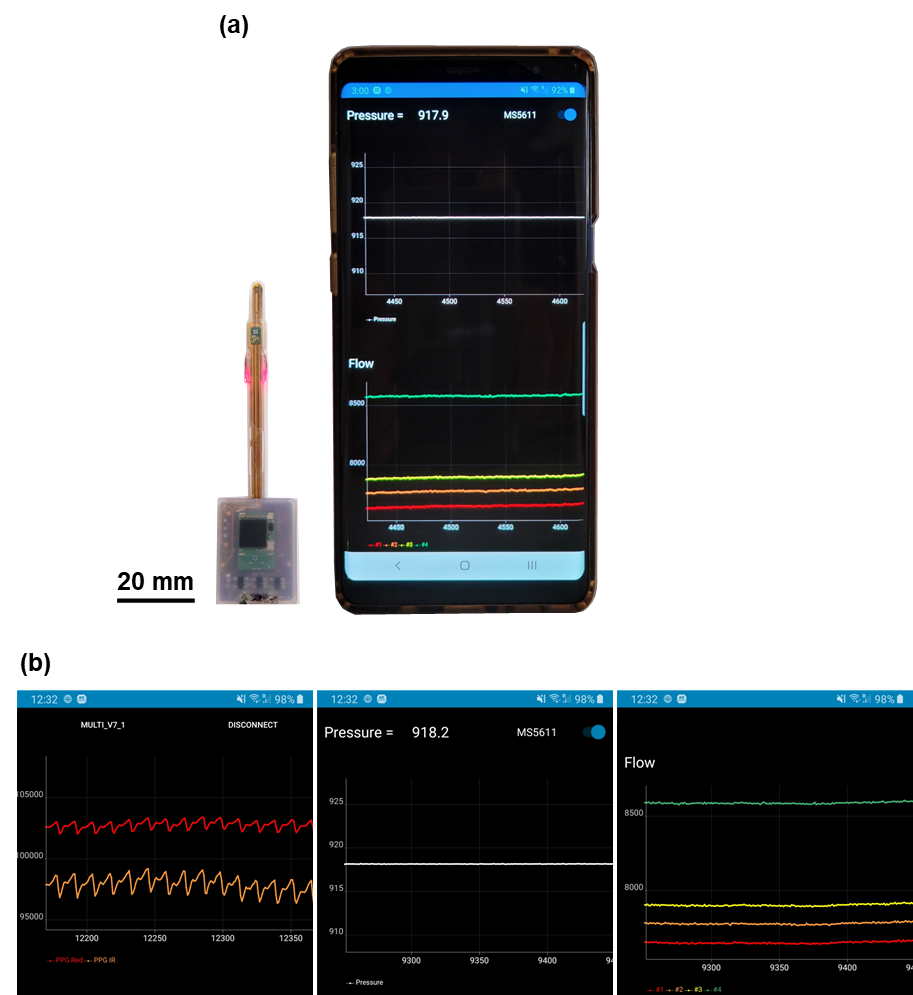
**

**Figure S2.** Multimodal probe and user interface. **(a)** Photograph of a cell phone to display real-time monitoring results by using a custom app and device. **(b)** Enlarged displays of simultaneous monitoring of StO_2_ (left), pressure (middle), and flow sensors (right).


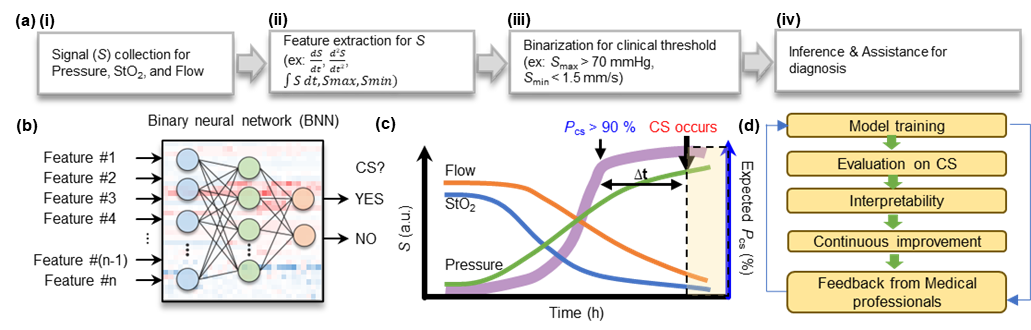


**Figure S3.** Envisioned procedure for multimodal signal collection, feature extraction, and neural network analysis to diagnose compartment syndrome (CS). **(a)** (i) signal collection for pressure, StO_2_, and flow, (ii) feature extraction for S, (iii) binarization for clinical threshold, (iv) inference and assistance for diagnosis, **(b)** binary neural network scheme for determination of real compartment syndrome, **(c)** example of scenarios for the expected probability for compartment syndrome on the basis of compartment syndrome, **(d)** flow chart for model training, evaluation on CS, and others in a feedback loop.

**(a)**

**
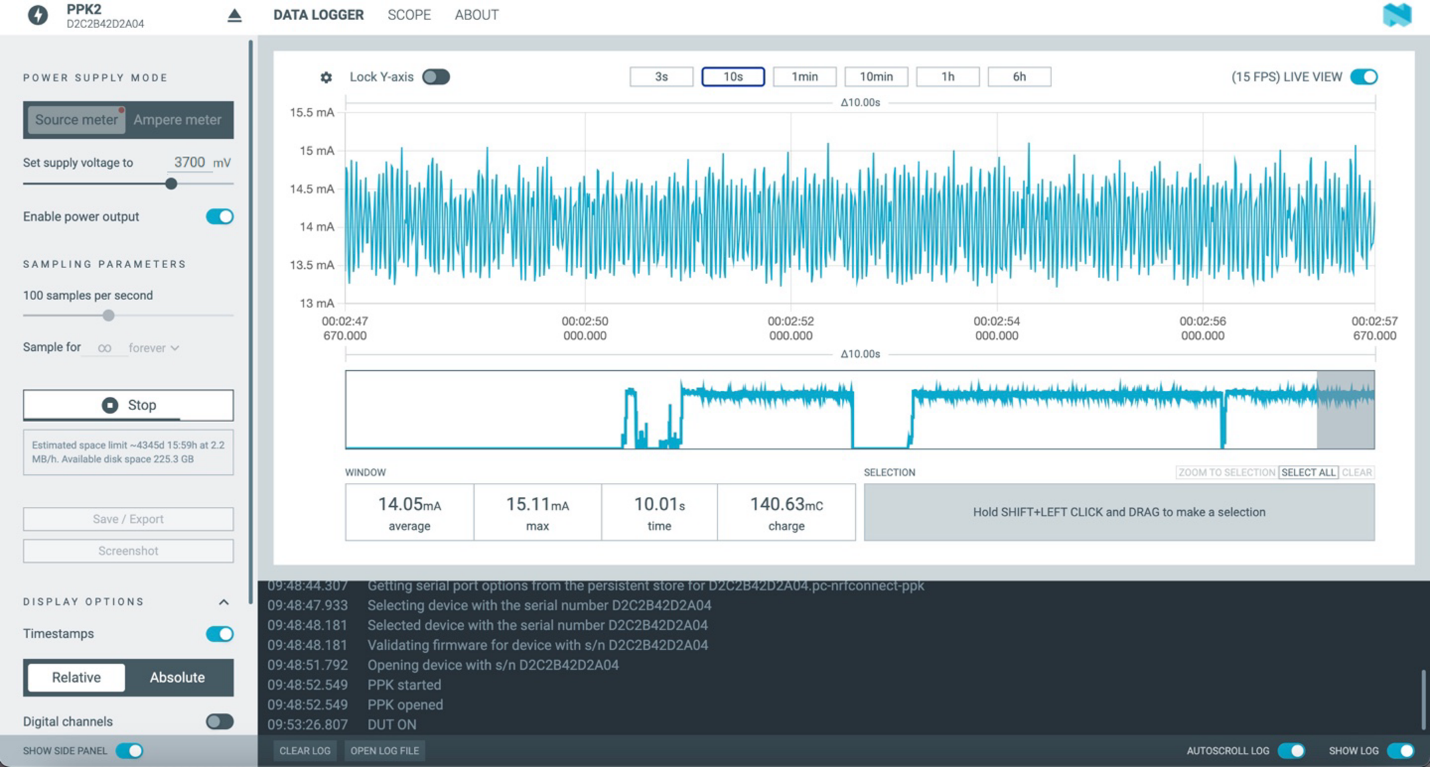
**

**(b)**

**
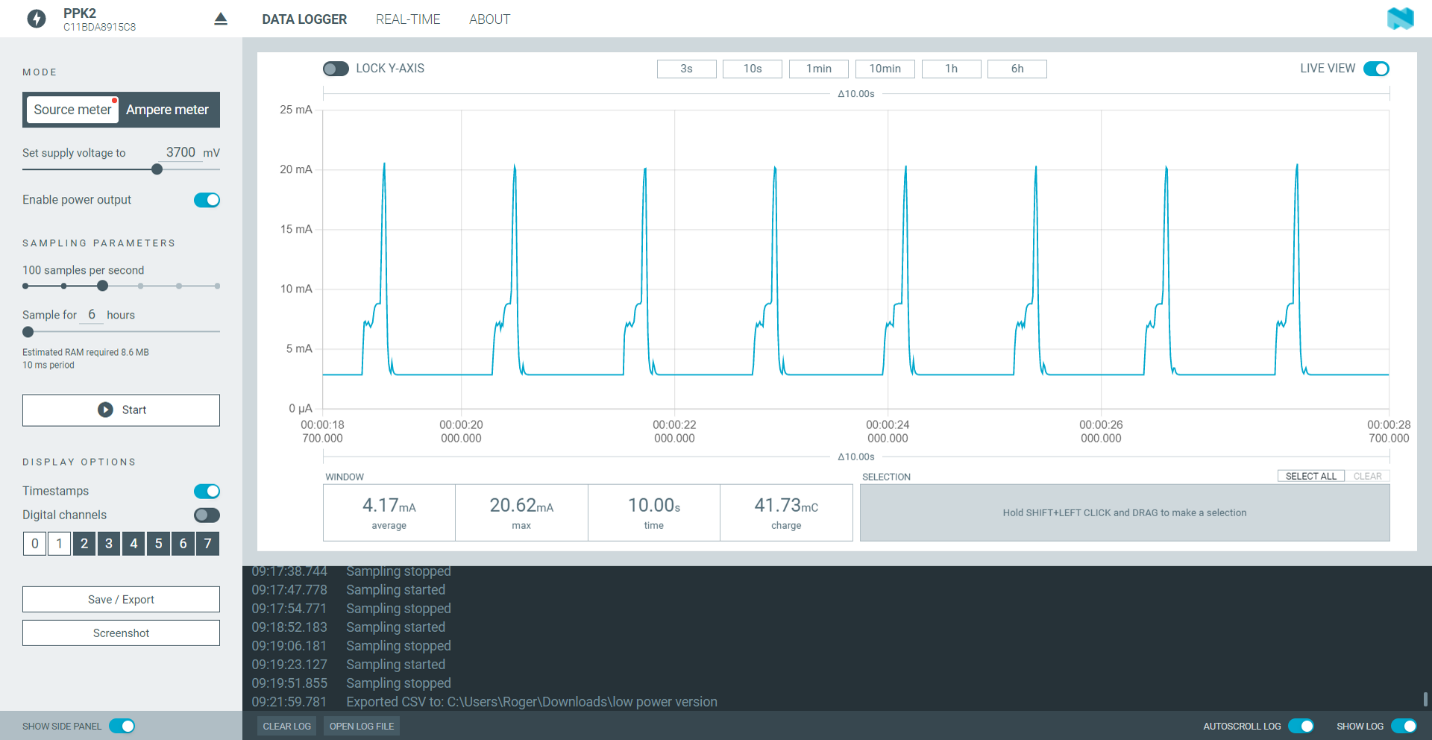
**

**Figure S4.**  Current consumption level during continuous operation with **(a)** 20Hz and **(b)** 1 Hz.

**
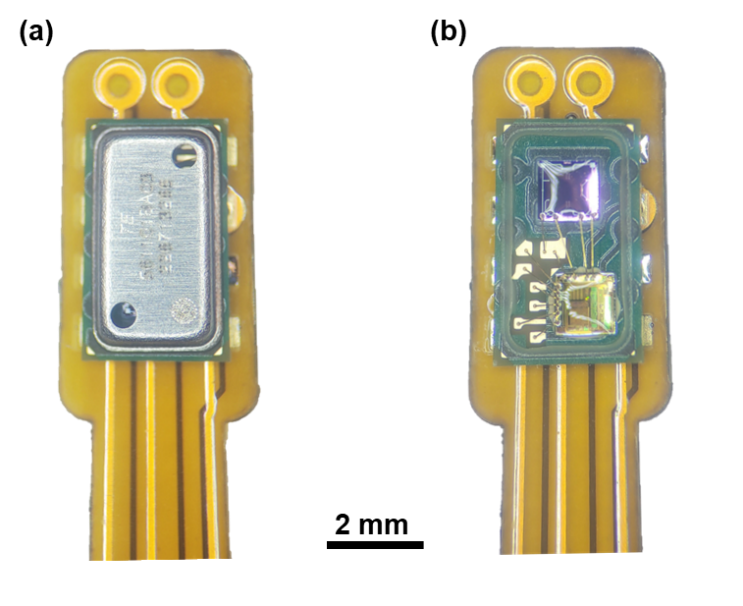
**

**Figure S5.** Photographs of pressure sensors on the FPCB. **(a)** Before and **(b)** after removing the metal cap.










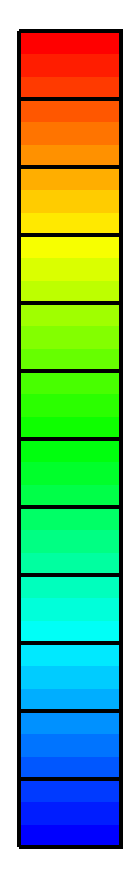




**0**

**10**

**20**

**30**

**40**

**50**

**60**

**70**

**80**

**90**

**100**

**110**

**120**

**Pressure (mmHg)**

**Applied pressure = 5 mmHg**

**Applied pressure = 30 mmHg**

**Applied pressure = 60 mmHg**

**Applied pressure = 70 mmHg**

**Applied pressure = 70 mmHg**

**(a)**

**(b)**

**(c)**

**Ranging from**

**68.9 ~ 70.5 mmHg**

**y**

**x**

**Figure S6.** Finite element analysis of pressure distribution. **(a)** Pressure distribution of the probe under applied pressures of 5, 30, 60, and 70 mmHg. **(b)** Pressure distribution on the surface of the sensor when 70 mmHg of pressure is applied. **(c)** Correlation between the applied pressure and the pressure measured on the surface of the sensor.

**
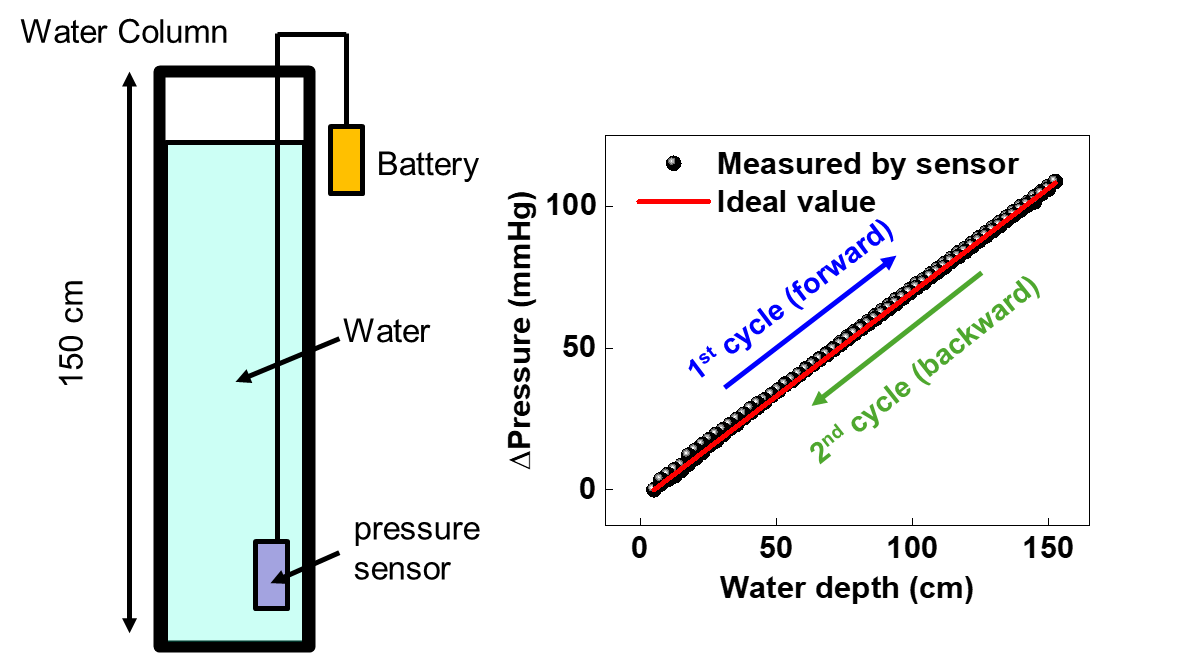
**

**Figure S7.** Measurement setup for pressure in a water column (left) and the corresponding measured pressure values (right).


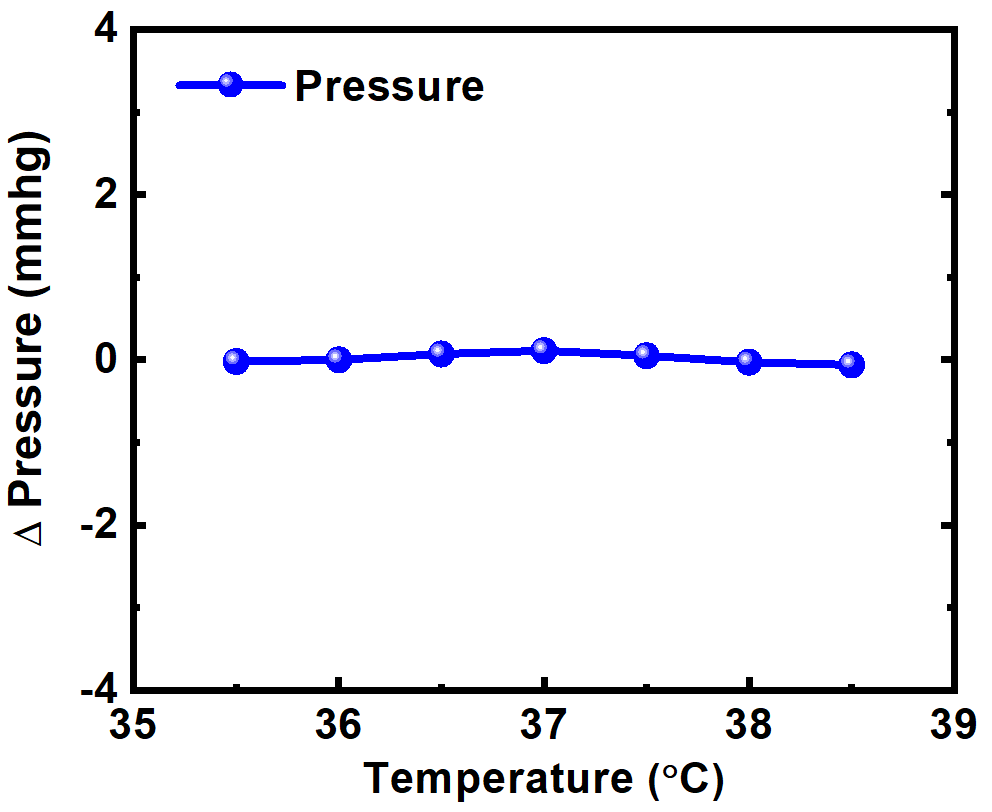


**Figure S8.** Experimentally measured pressure variations of a multimodal sensor probe within the body temperature range

**
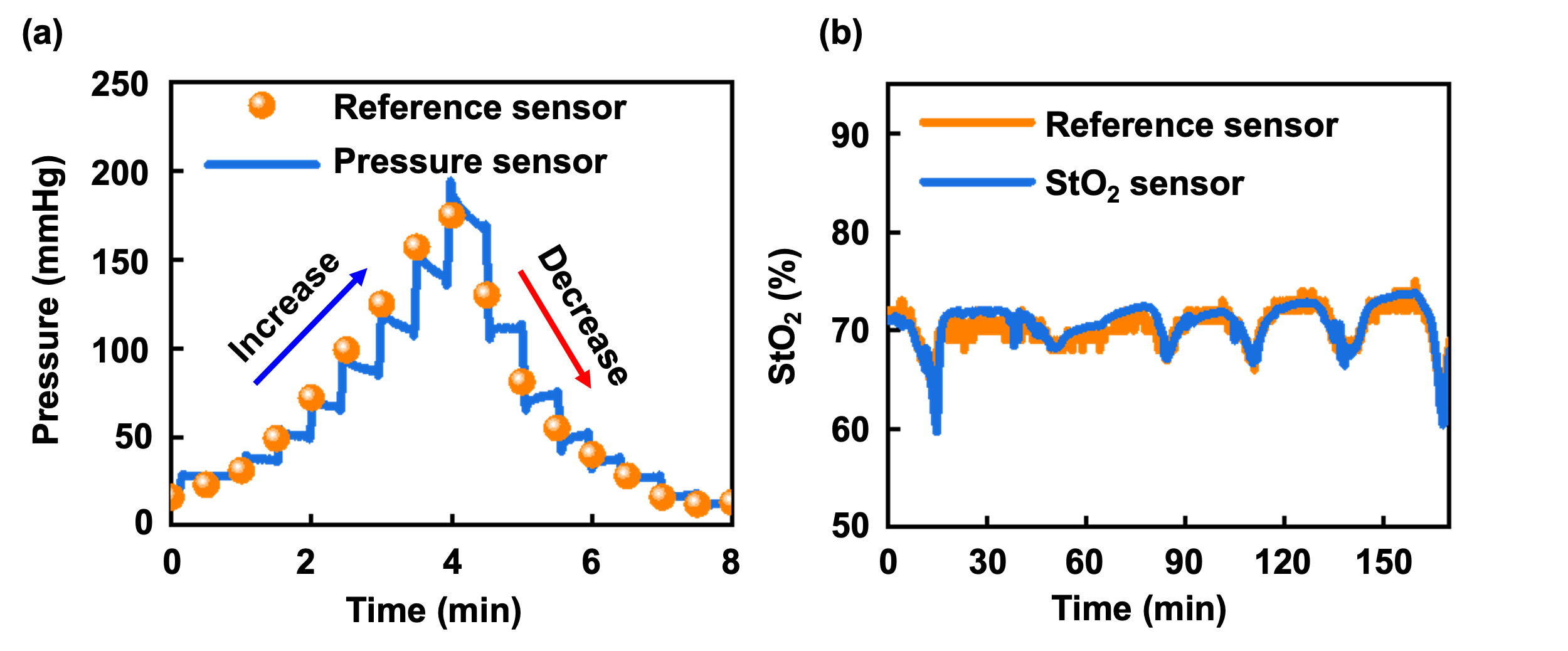
**

**Figure S9.** Preliminary feasibility studies. **(a)** Pressure and **(b)** StO_2_ values in the tissue of porcine model measured by multimodal probe (blue line) and reference pressure sensors (Phillips Intellivue) and StO_2_ sensor (Vioptix). The data demonstrates implanted multimodal probe has high sensitivity consistent with commercial sensors.

**Figure S10.** Temperature differences (ΔT_14_) between thermistor 1 and thermistor 4 at environmental temperatures of 35°C, 37.5°C , and 40°C under constant water flow.


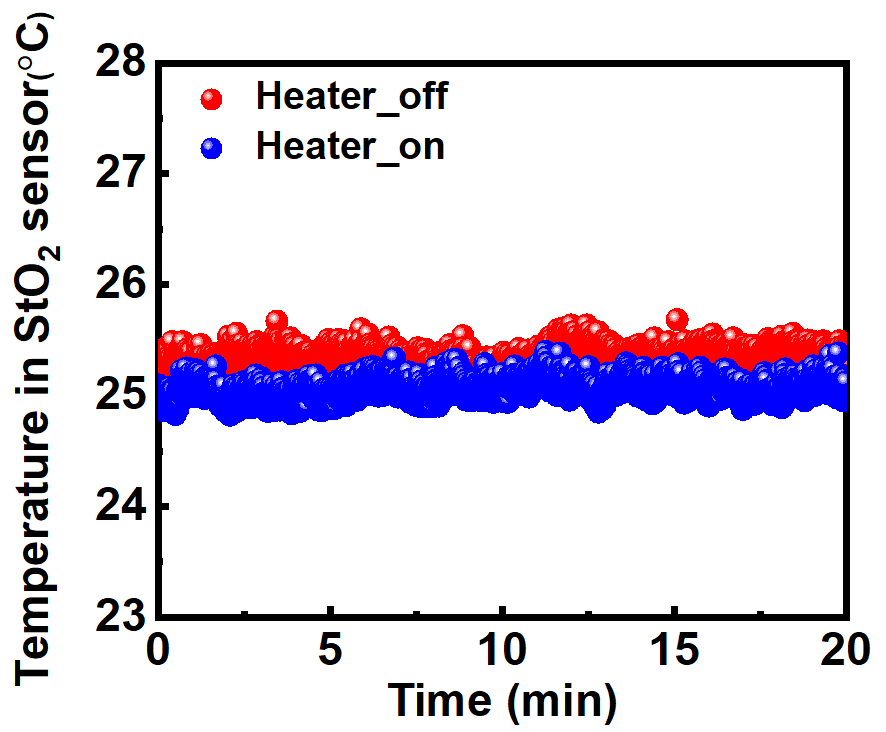


**Figure S11.** Time-dependent temperature profiles measured in the area of the StO_2_ sensor when the heater turns off and on.


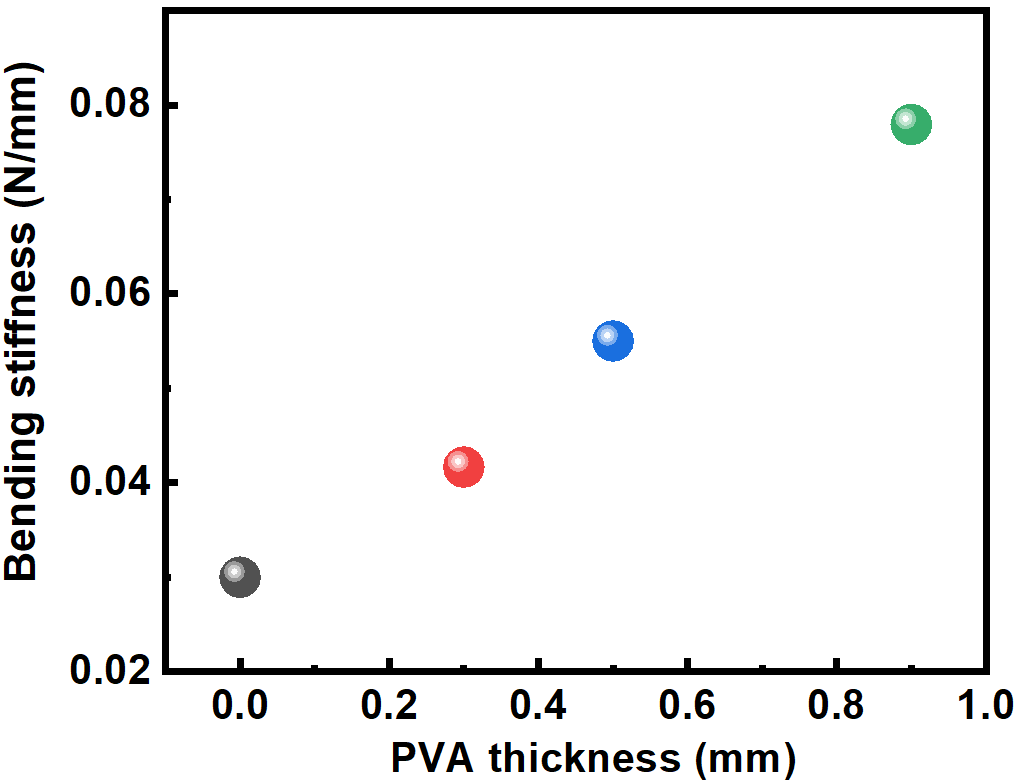


**Figure S12.** Bending stiffness of multimodal probe with a PVA coating.


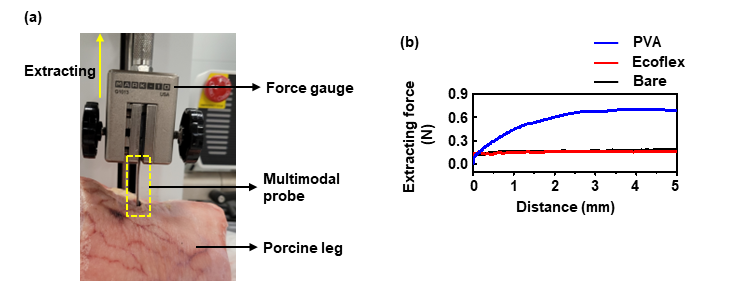


**Figure S13.** Mechanical stability of multimodal probe in the tissue. **(a)** Measurement set up and **(b)** extracting force of the bare, Ecoflex-coated, and PVA-coated probe from the tissue.

**
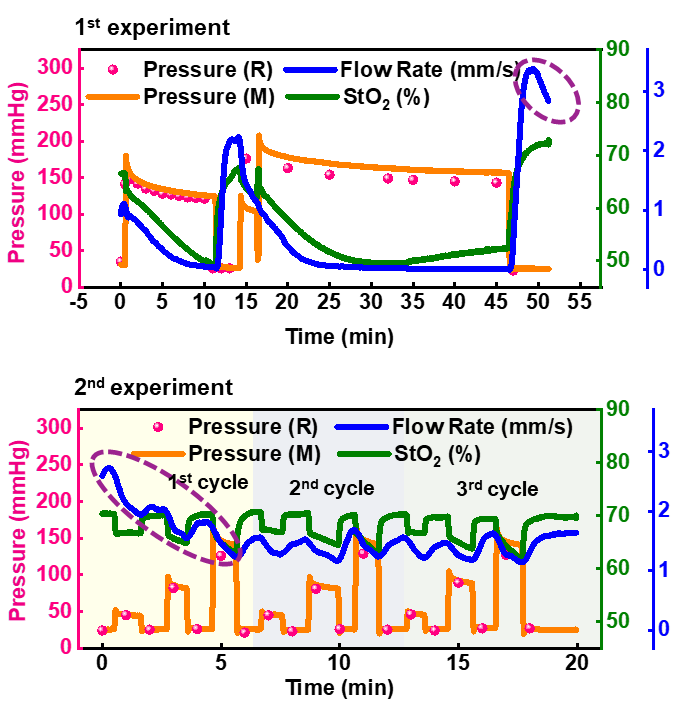
**

**Figure S14.** A representative data set for two experiments to explain the decreasing trend of flow rate in the 1st cycle of the short-term experiment.

The initial cycle of an increase in pressure leads to a gradual decrease in flow rate, likely affected by the process of thermal equilibration of the probe with the surrounding tissue. Figure S6 presents a representative set of tests from two consecutive cycles: the first trial (upper figure) lasting 50 min, followed by a 1 min trial (bottom figure). After conducting a 50 min-term experiment with the same device, a recovery in flow rate around 45–50 min overshoots the baseline before stabilizing at 2 mm/s (as highlighted by the purple dot-circle in the upper figure of Figure S14). This behavior indicates dynamic interactions within the tissue during the recovery phase. A decreasing trend observed after 50 min in the 1^st^ experiment occurs also during the first cycle of the 2^nd^ short-term experiment, as shown in the bottom figure (purple dot-circle in Figure S14). This similarity is likely because the second experiment was conducted immediately after the first. This behavior indicates dynamic interactions within the tissue during the recovery phase.


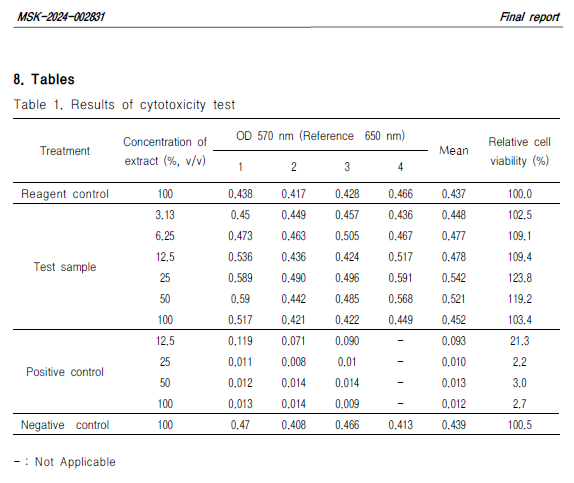


**Figure S15**. Cytotoxicity test report.


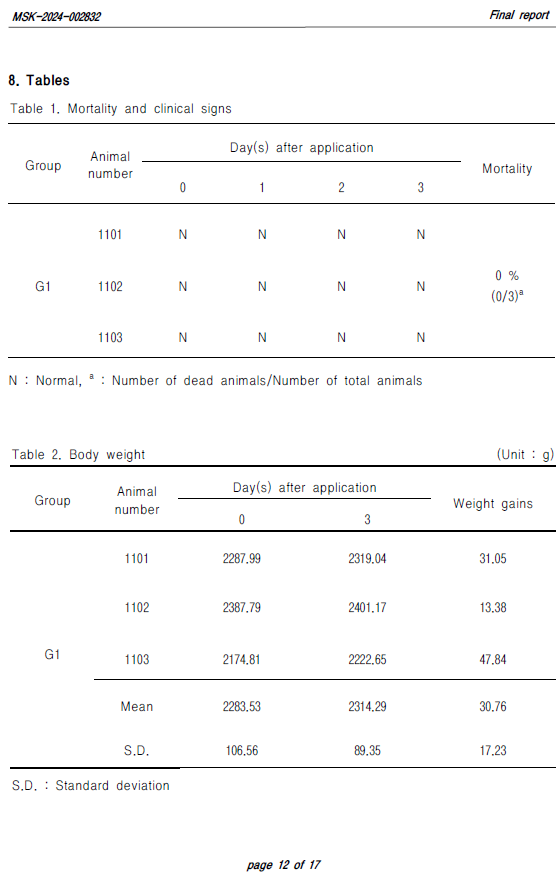


**Figure S16.** Intracutaneous reactivity test report (1/2).


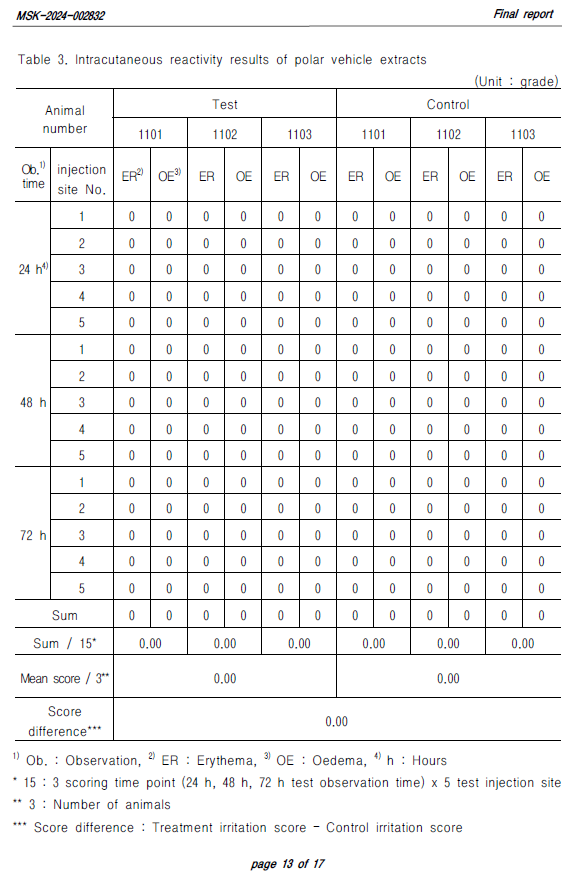


**Figure S17.** Intracutaneous reactivity test report (2/2).


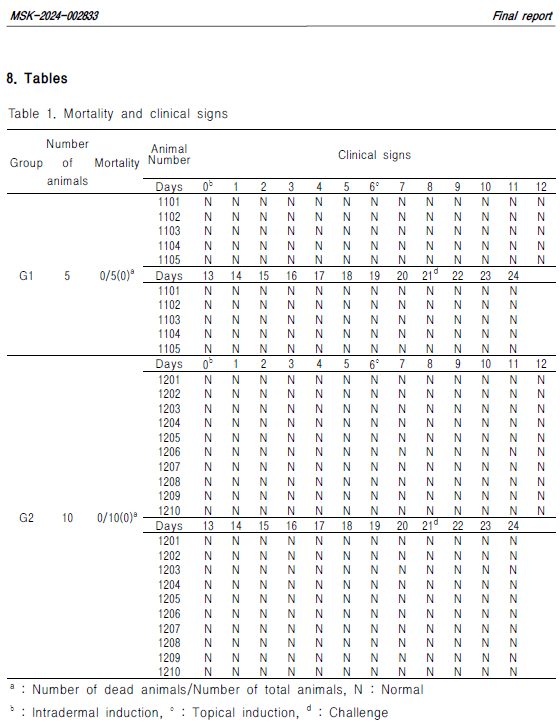


**Figure S18.** Skin sensitization test report (1/5).


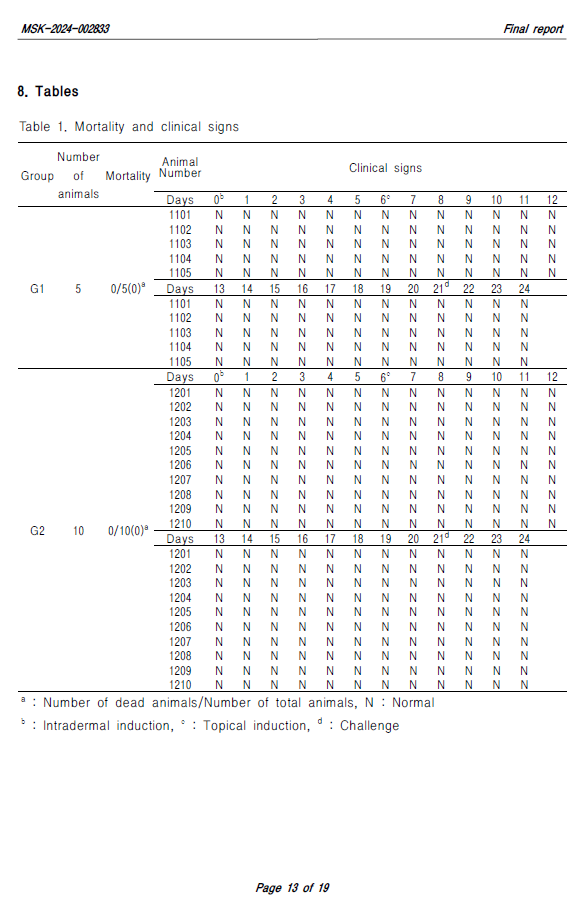


**Figure S19.** Skin sensitization test report (2/5).


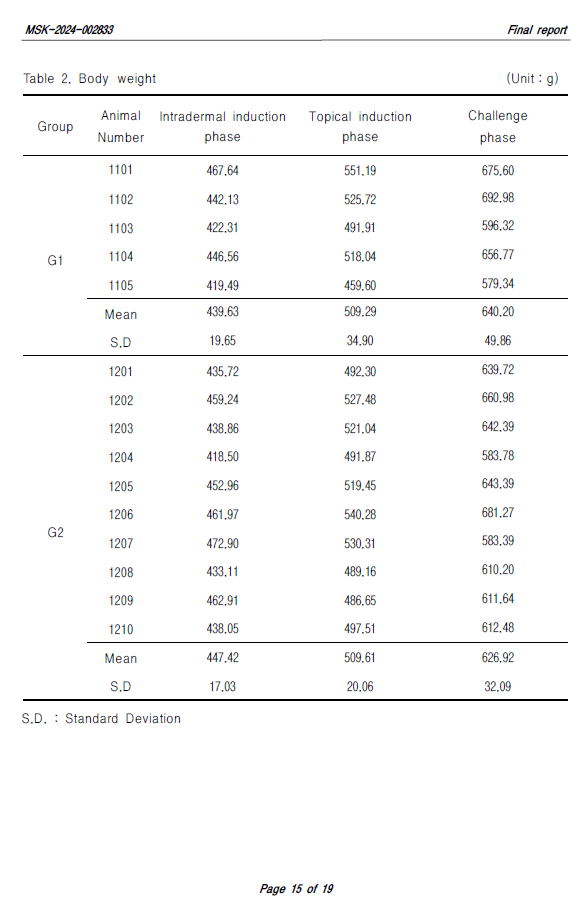


**Figure S20.** Skin sensitization test report (3/5).


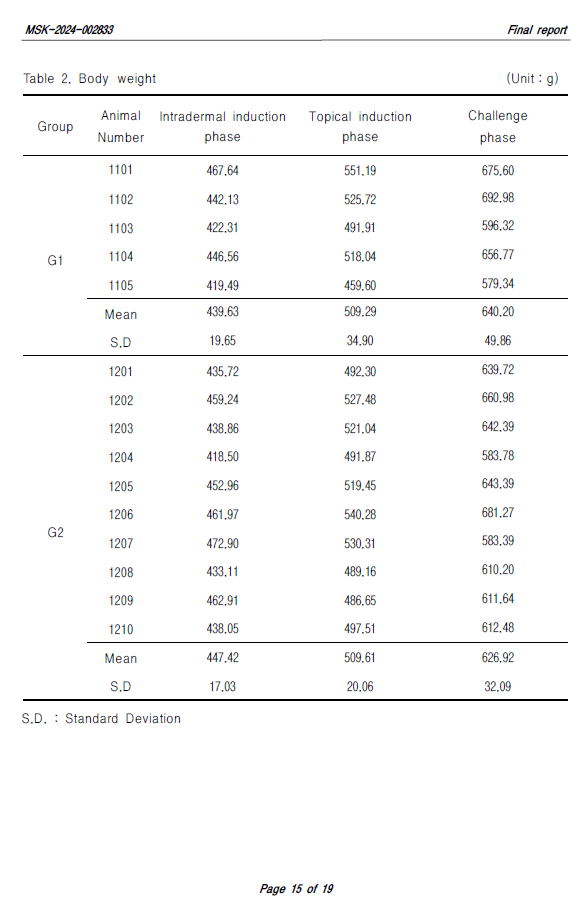


**Figure S21.** Skin sensitization test report (4/5).


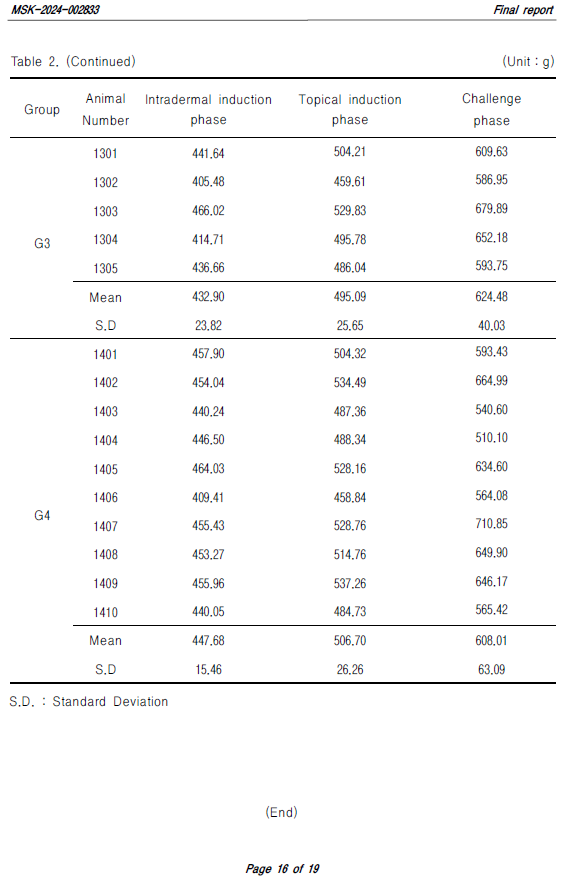


**Figure S22.** Skin sensitization test report (5/5).


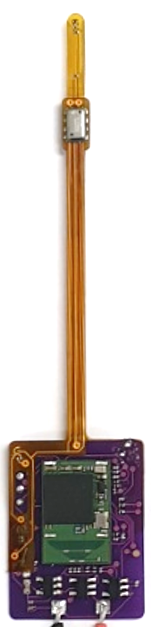

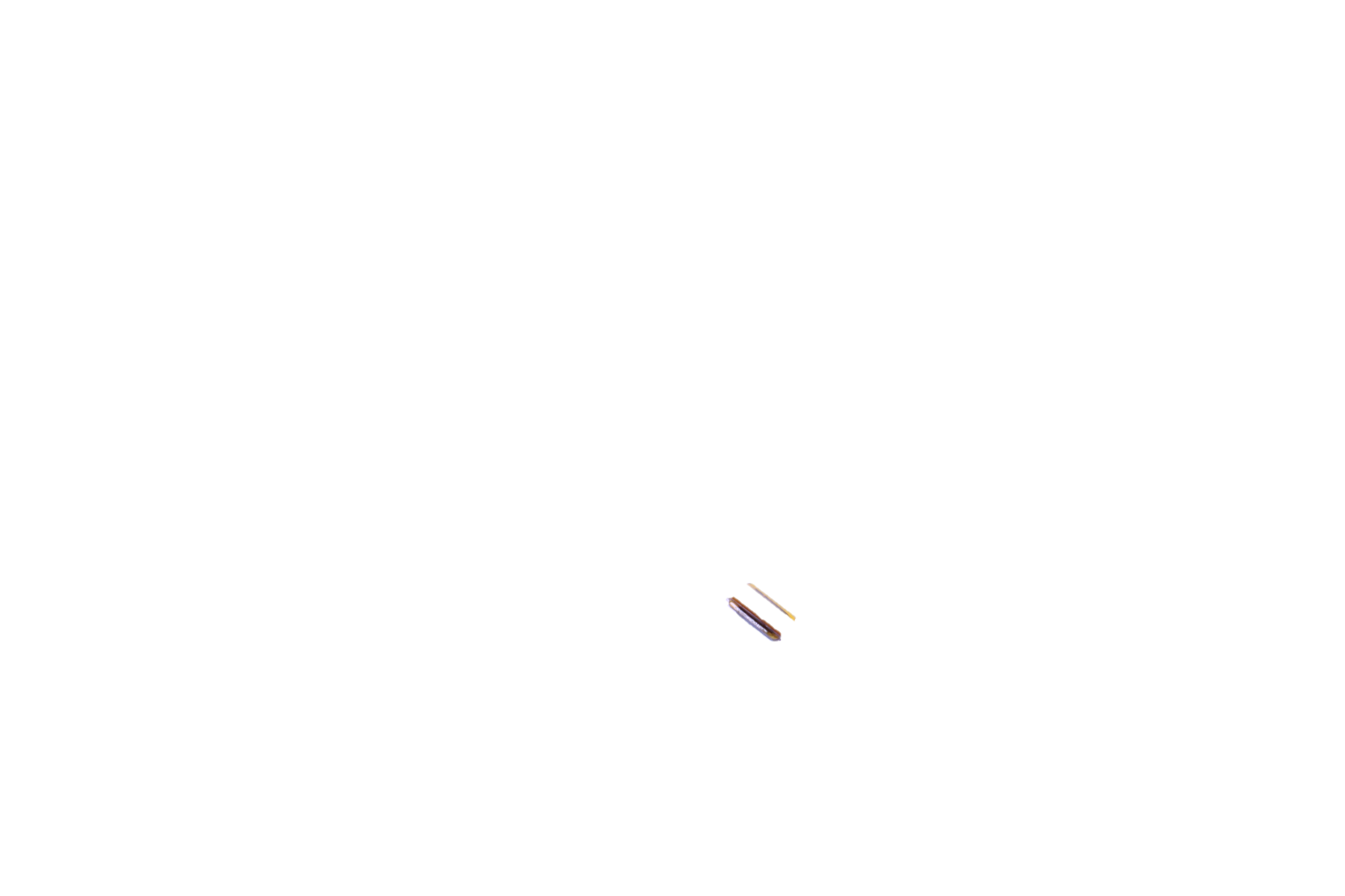

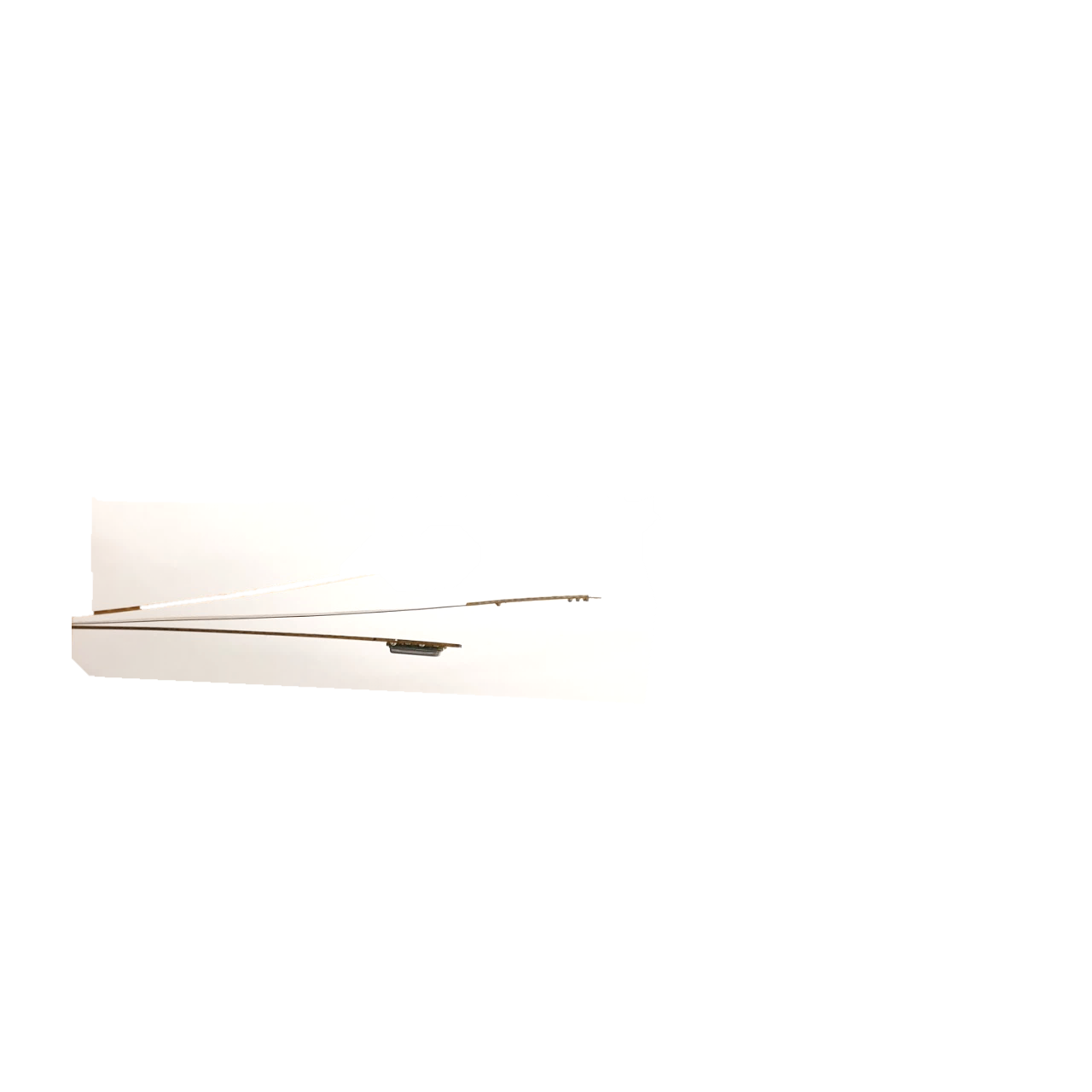

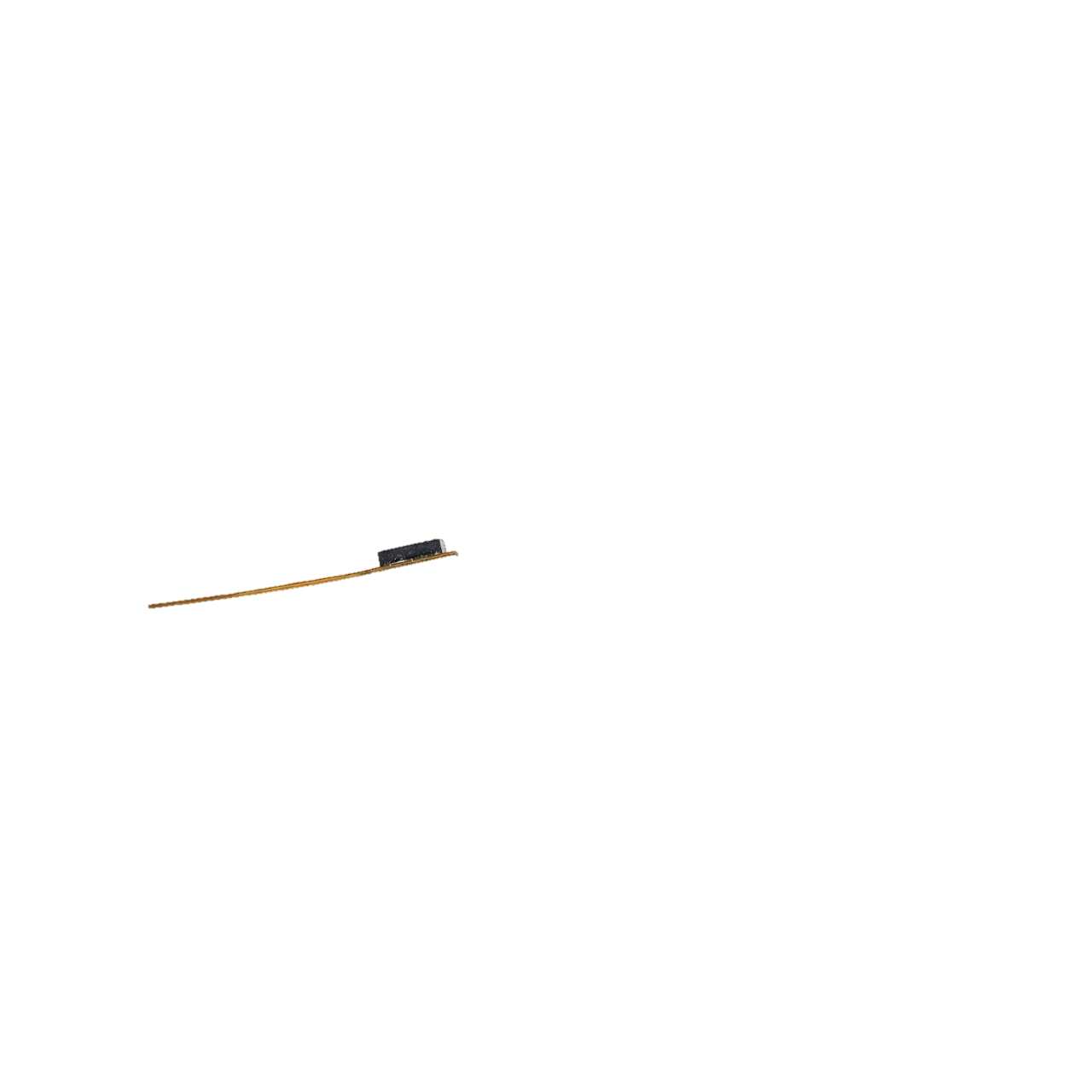

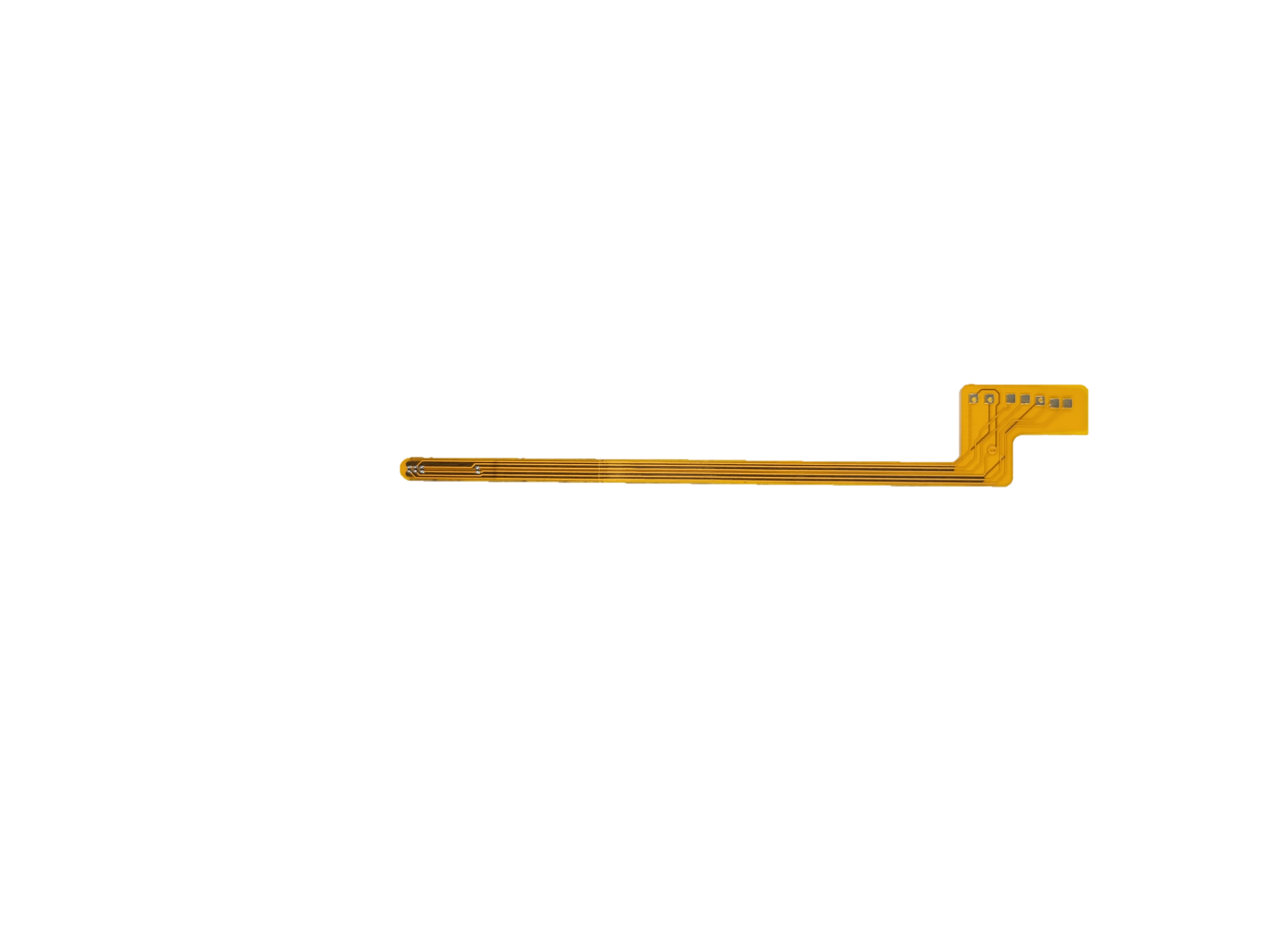

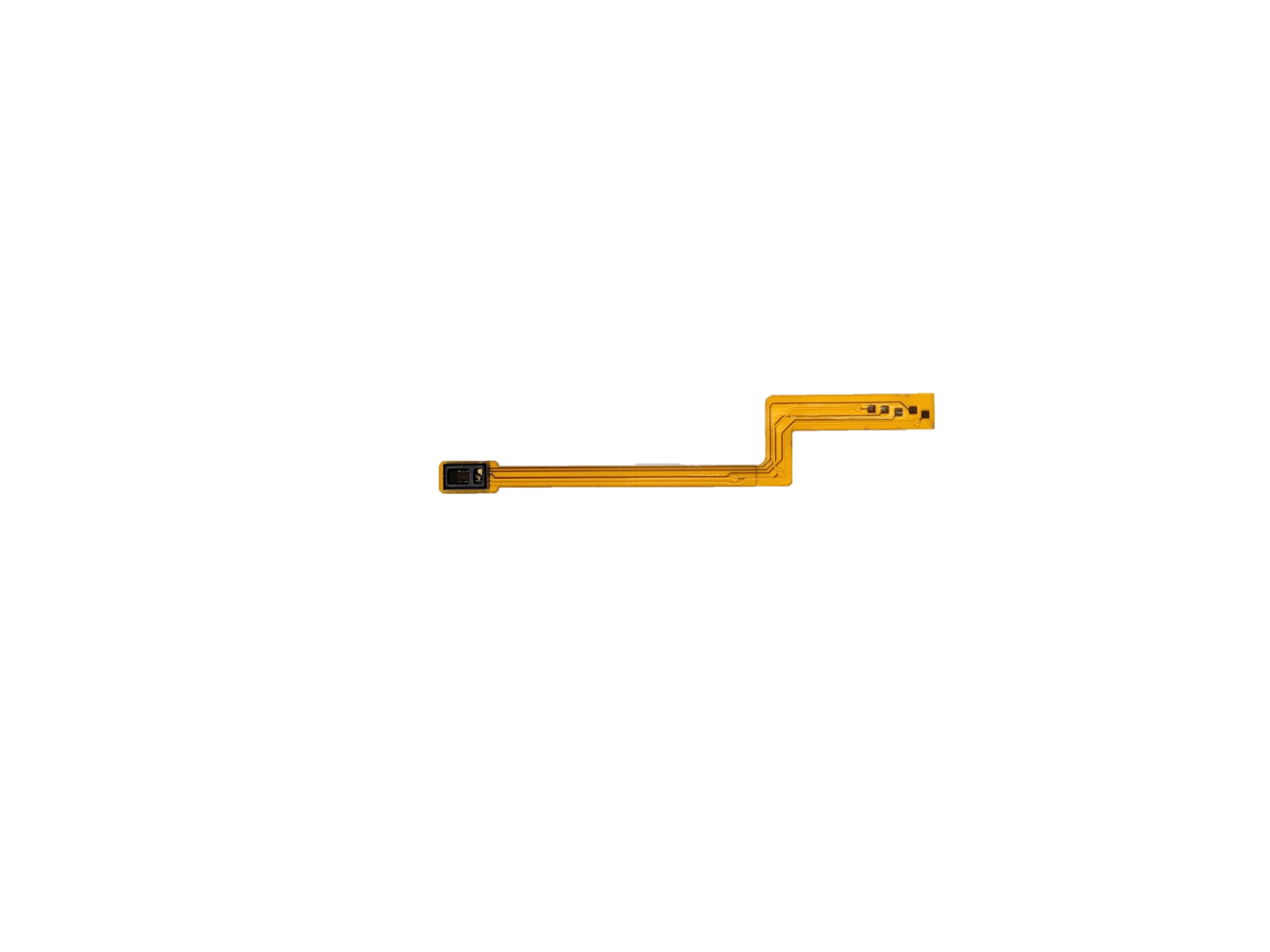

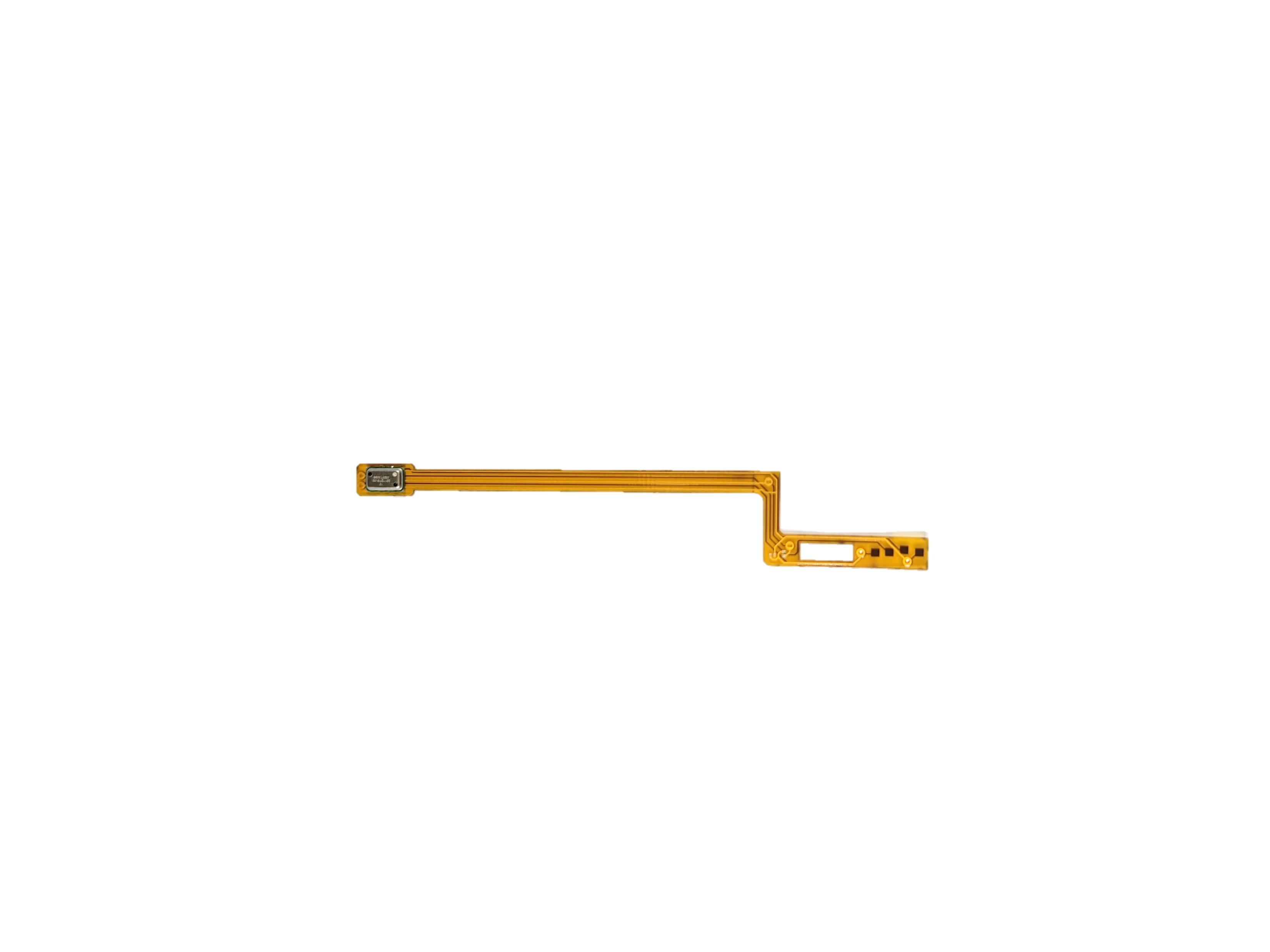


**(a)**

**Flow sensor**

**10 mm**

**StO_2_ sensor**

**Pressure sensor**

**Pressure**

**StO_2_**

**Flow**

**Flexible Substrate**

**Multimodal probe**

**Main controller**

**Signal line**

**NINA B3 module**

**LDOs and battery line**

**(b)**

**Contact pads with main controller**

**Figure S23.** Photographs of device configuration. (a) Individual sensors and (b) the assembled multimodal probe with the main controller.

**
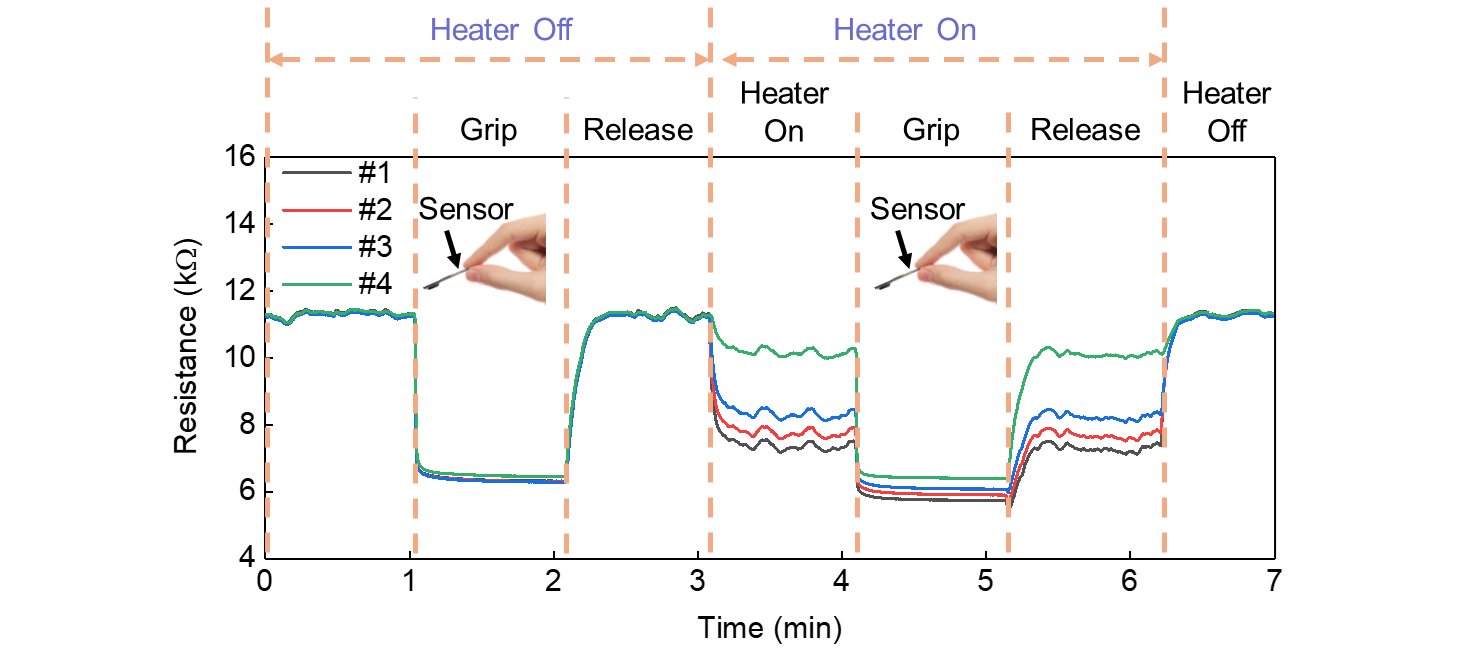
**

**Figure S24.** Variations in the resistances of thermistors resulting from contact with a heater and a commercial human skin model(Suture Pad, Simskin), which was pre-warmed to 36°C in an oven.
